# Polymer Crystallization: Universal Macroscopic Description via the Autocatalytic Hoffman–Lauritzen Approach

**DOI:** 10.1021/acsomega.5c00067

**Published:** 2025-04-20

**Authors:** Roman Svoboda, Jana Machotová

**Affiliations:** † Department of Physical Chemistry, Faculty of Chemical Technology, University of Pardubice, Studentská 573, Pardubice 532 10, Czech Republic; ‡ Institute of Chemistry and Technology of Macromolecular Materials, Faculty of Chemical Technology, University of Pardubice, Studentská 573, Pardubice 532 10, Czech Republic

## Abstract

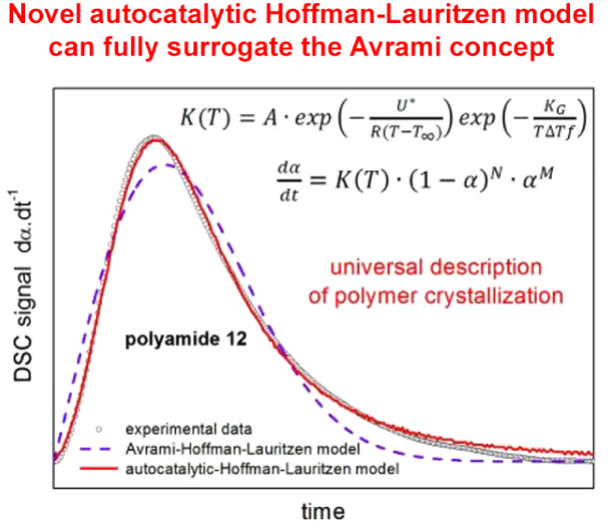

For the description of the crystallization in polymers, the exponential autocatalytic *N*^th^-order model, paired with the Hoffman–Lauritzen nucleation–growth theory (the conjunction denoted as the MCHL model), was introduced as an alternative to the classical Avrami concept. It was demonstrated, employing theoretical simulations, that the Avrami-based kinetics exhibits a strictly defined interval of asymmetries for the derivative kinetic data (conformable to the differential scanning calorimetry crystallization peaks), often incompatible with the experimental observations. On the other hand, the MCHL model is highly flexible, reproducing and largely extending the range of the Avrami asymmetries, thus effectively covering all possible single-process manifestations of the polymer crystallization mechanisms. The MCHL model was further proven to comply with the *t*_0.5_-based evaluation of the Hoffman–Lauritzen nucleation constant, reducing the number of variables in the nonlinear optimization-based enumeration of the model’s parameters. A unique relationship between the Avrami and MCHL kinetic was found.

## 1 Introduction

In polymers, crystallization is a process by which the polymer chains organize into a highly ordered structure. The process is initiated by nucleation, which can be either homogeneous or heterogeneous. Homogeneous nucleation occurs spontaneously and uniformly throughout the polymer melt or solution without any preferential nucleation sites; it requires overcoming a high energy barrier because it relies solely on the thermal fluctuations within the polymer matrix to form stable nuclei (as such, it is relatively rare). On the other hand, heterogeneous nucleation occurs at specific sites such as impurities, fillers, additives, or preexisting surfaces within the polymer matrix, where the presence of these sites reduces the energy barrier for nucleation, making it easier for the nuclei to form. The temperature dependence of the nucleation rate generally depends on the degree of undercooling, internal self-diffusion of the polymer chains (associated with the viscosity), presence of the nucleating agents or preferred sites, polymer structure itself (molecular weight and configuration of chains), and external/processing forces causing alignment of the polymer chains.^1−3^

For the crystal growth (the process consequent to the initial nucleation) in polymers, the key process is the chain folding into thin plate-like structures, i.e., lamellae, with ∼10 – 20 nm thickness. The growth of the lamellae proceeds dominantly at the crystal faces, where additional polymer chains can be incorporated into the lamellae. The most common supramolecular structure formed from the lamellae is a spherulite, a spherical aggregate of lamellae radially growing outward from a central nucleation point. The branching and impingement of the lamellae within each spherulite do not lead to a full infilling of the whole volume of the spherulites, leaving a certain portion of the space between the lamellae in an amorphous state—hence, practically no polymer can reach 100% crystallinity. Similarly, the impingement of individual spherulites also leads to the prevalence of the amorphous phase or imperfect crystalline structures at the impingement interface (crystal grain boundaries). The primary crystal growth can be further followed by lamellar thickening (reorganization associated either with partial melting or with prolonged thermal annealing at temperatures high enough for the chain mobility to be sufficient for refolding into more stable, thicker lamellar structures), postcrystallization growth (additional polymer chains crystallizing onto existing lamellae due to annealing at temperatures close to the original crystallization temperature) or secondary crystallization (where new nuclei form in the vicinity of the existing crystalline regions, often driven by the reduction of the free energy associated with the newly formed internal-stress-induced active sites).^1,4−6^

Since a macroscopic manifestation of the overall crystallization extent (as opposed to the microscopically observed spherulitic growth rate) is technologically much more important as well as experimentally easier recorded (using differential scanning calorimetry, DSC), a major effort has been focused on the macroscopic crystallization models. For polymeric materials, this field is dominated by the combination of the Avrami mathematical framework^7−11^ with the Hoffman–Lauritzen nucleation–growth theory.^12^ The Avrami model is based on the extended volume concept—a theoretical volume occupied by the newly formed crystalline phase if the entire sample was still fully amorphous (i.e., with no impingement and overlapping effects in place). The true volume of the newly formed crystalline phase is then considered to be proportional to the remaining volume of the amorphous (untransformed) fraction, which leads to eq 1:
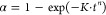
1where α is the degree of conversion from amorphous to crystalline phase, *K* is the rate constant (comprising the growth rate *Ġ* and nucleation rate *Ṅ*), *t* is time and *n* is the Avrami kinetic exponent which reflects different initial conditions for nucleation and geometry of the crystallites (comprehensive summary of potential values of *n* is given in refs^13,14^.

The temperature-dependent *K* is then for the polymeric materials usually expressed in terms of the Hoffman–Lauritzen theory (eq 2):
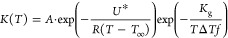
2where *A* is a pre-exponential factor (essentially translating the manifestation of the exponential dependences to the macroscopic level), U* is the activation energy for the segmental movements of the polymer chains (considered to be 6,300 J·mol^–1^ for the majority of polymeric materials^12^), *R* is the universal gas constant (8.314 J·K^–1^·mol^–1^), *T*_∞_ is the temperature under which all motions associated with a viscous flow are supposed to be significantly longer than the given experimental time scale for the crystal growth (customarily *T*_∞_ = *T*_g_ – 30 °C, where *T*_g_ is the glass transition temperature), Δ*T* is the undercooling (defined as Δ*T* = *T*_m_^eq^ – *T*, where *T*_m_^eq^ is the equilibrium melting temperature), and *f* is the correction factor defined as *f* = 2*T*/(*T*_m_^eq^ + *T*), and *K*_g_ is the Hoffman–Lauritzen nucleation constant (the kinetic parameter associated with nucleation) defined as (eq 3):
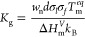
3where *w*_n_ is a constant associated with the nucleation regime, *d* is the thickness of a lamellar monolayer, σ_l_, and σ_f_ are the lateral/lamellae and chain fold free surface energies, respectively, *k*_B_ is the Boltzmann constant, and Δ*H*_m_*^V^* is the volumetric equilibrium melting enthalpy. Note that various other *K* expressions can be used instead of eq 2; for example, the crystallization from the glassy state is often described via implementation of the Arrhenius rate constant into eq 1. It is also worth noting that the term “rate constant” makes sense only for the isothermal data, as *K* is almost always a function of temperature and thus not constant during the nonisothermal treatment.

Due to the large importance of the polymer physics field and the huge variety of polymeric materials, many alternative models have been developed to accommodate the specific features of the observed crystallization behaviors. Starting with the Hoffman–Lauritzen theory, apart from several modifications of the theory itself,^15−21^ one of the most akin models is the Sadler-Gilmer model^22−24^ which bases the derivations on the entropic barrier instead of the enthalpic one (this distinct approach, however, leads to similar growth kinetics). In Hikosaka’s sliding diffusion model,^25−28^ the self-diffusion of a polymer chain along its chain axis direction extends the Hoffman–Lauritzen theory for the case of extended-chain crystals, where a new two-dimensional growth accounting for the specific anisotropic polymer topology occurs in addition to the standard lateral growth. Revision of the Hoffman–Lauritzen theory concerning the influence of the weight-average molecular weight (*M*_w_) on the nucleation process was introduced in Wunderlich’s molecular nucleation theory,^29−31^ which considers completion between the polymer fractionation (thermodynamic term favoring longer chains) and segregation (kinetic term favoring shorter chains). This concept was further developed and quantified in Hu’s intramolecular nucleation theory^32,33^ by employing a competition between the nucleation (*M*_w_-independent) and melting (*M*_w_-dependent) free energy barriers, the occurrence of the *M*_w_-dependent molecular segregation process can be explained. One of the most advanced approaches to polymer nucleation is represented by Muthukumar’s continuum theory,^34^ where a unified formalism interconnects the nucleation-controlled and diffusion-controlled crystal growth rates.^35,36^ In this approach, a dense boundary layer (adjacent to the crystal growth front) is associated with an entropic barrier adjusted for the *M*_w_-dependent crystal growth; the temperature-dependent crystal growth rate *G*(*T*) can then be expressed as a multiplication of the terms corresponding to the two kinetic regimes (nucleation and diffusion) (eq 4):
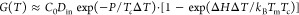
4where *C*_0_ is the initial polymer concentration, *D*_in_ is the diffusion coefficient inside the boundary layer, *T*_c_ is the crystallization temperature, and *P* is a parameter depending on the melting enthalpy (Δ*H*_m_), the melting temperature (*T*_m_), *T*_c_, and the entropic barrier. It should be noted that all the above-mentioned theories (including the Hoffman–Lauritzen) are kinetic, introducing a competition between the driving force and a nucleation barrier. As such, being incorporated into *K* from eq 1, they provide an akin description of the experimental macroscopic crystallization data. Contrary to the microscopic nucleation–growth theories, the macroscopic description of the polymer crystallization data (usually in its derivative form, i.e., dΔ*H*/d*T*, measured by DSC) largely relies on the Avrami concept or its variations, with certain solutions being adopted even from distant fields dealing with metallic glasses or construction materials.^37−48^ However, despite the numerous modifications, the similarity of the intrinsic concept of these models leads to the limitation of their flexibility in the description of the real crystallization data.

**Figure 1:**
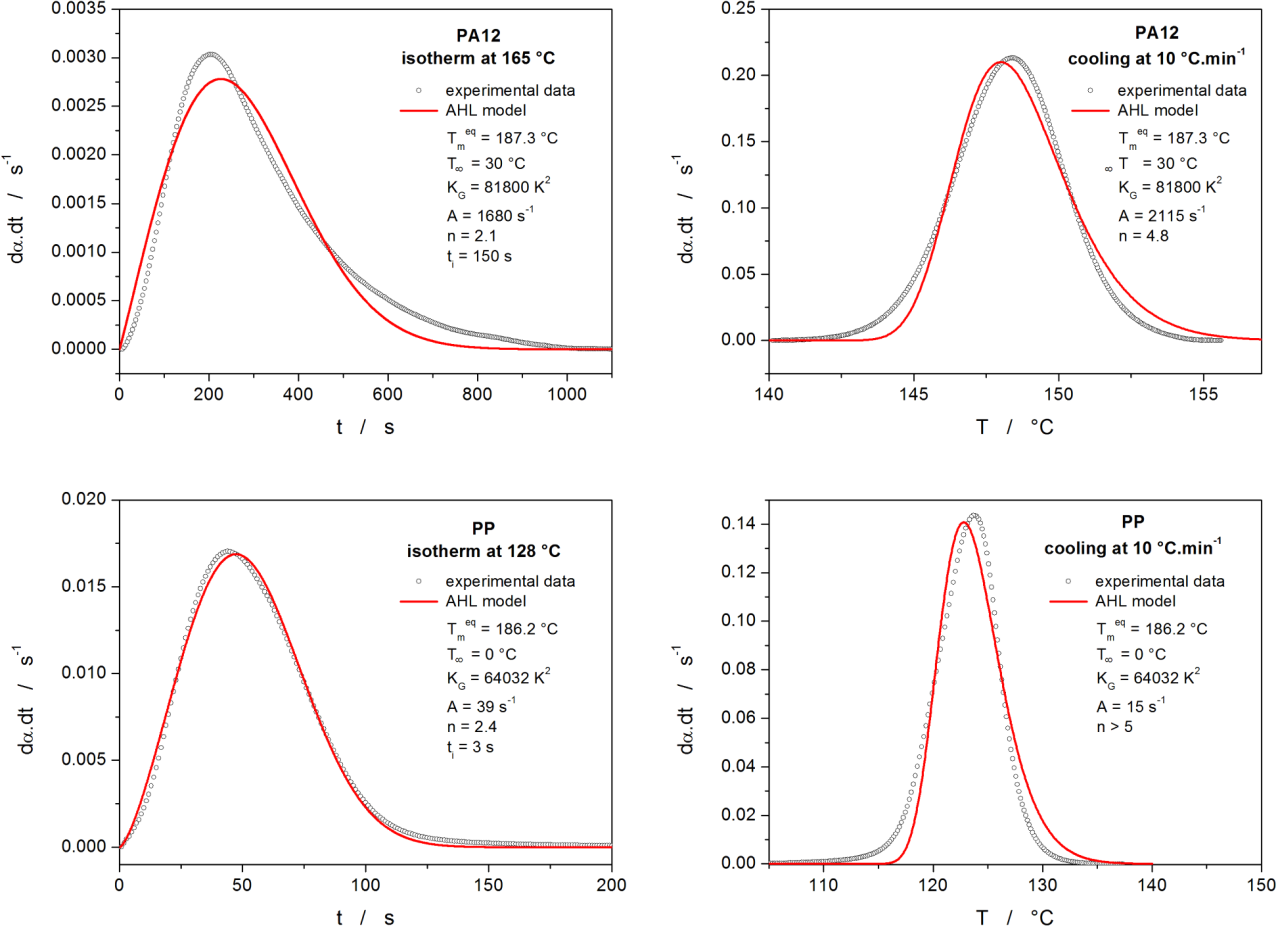
Examples of the isothermal and nonisothermal crystallization data obtained for polyamide 12 (PA12) and isotactic polypropylene (PP) fit by the AHL model (combination of eqs 1 and 2). The parameters of the best fits are displayed in the respective legends in each graph.

Several examples of these inaccuracies, in this case associated with the restricted description of the DSC crystallization data in terms of the combined Avrami-Hoffman–Lauritzen theories, are shown in Figure 1. For the isothermal data, eq 2 can be implemented into eq 1 to directly calculate the α-*t* dependence, as *T* = const. in such case. For the nonisothermal data, the running integral of *K*(*T*) replaces *K* in eq 1—see the Supporting Information material for the corresponding expression. One can see that a typical systematic deviation, clearly associated with the characteristic asymmetry of the Avrami-Hoffman–Lauritzen (AHL) model (eqs 1 and 2), occurs between the model and experimental data obtained for commercial-grade polyamide 12 (PA12, trade name PA 2200, EOS GmbH, Germany) and commercial-grade isotactic polypropylene (PP, trade name BE52, Borealis Polyolefine GmbH, Austria). It can be shown that the deviations are inherent to the model and not associated with potential thermal gradients in the sample or on the sample/sensor thermal path (which could produce similar experimental signals) because the same deviations occurred in the case of the PA12 material even at a cooling rate (*q*^–^) of 0.1 °C·min^–1^. Moreover, the extent of the deviations can be temperature/regime dependent (which results from the comparison of the PA12 and PP materials, where the isotherm for PA12 was performed at *T* ≈ *T*_m_ – 10 °C, whereas the PP isotherm was realized at *T* ≈ *T*_m_ – 40 °C). In addition, the deviation between the model and the experimental data further distorts the results by leading to overestimated *n* values without physical meaning.

It is obvious from Figure 1 that for the depicted data, the AHL fits do not reflect (to a lesser or greater extent) the physicochemical reality of the crystallization process. Thus, the advantage of the Avrami equation being derived on a physically meaningful basis is lost due to the corresponding (distorted) *n* not representing the real nucleation or growth conditions. Correspondingly, any kinetic predictions (which are the main purpose of performing the kinetic analysis and modeling the experimental data) based on such inaccurate descriptions of the experimental data are meaningless. In such a situation, a completely distinct concept is needed, with the primary goal of an accurate description of the experimental data and only the secondary aim of interpretation of the model parameters. In the present paper, such a concept will be introduced. In particular, the first part of the paper will be dedicated to the identification of the performance boundaries and limitations of the AHL model (employing theoretical simulations) so that the possible sources of errors can be considered before deciding to employ the novel concept. In the second part of the paper, a new concept will be introduced based on autocatalytic crystal growth combined with the Hoffman–Lauritzen nucleation–growth theory.

## 2 Results and Discussion

### 2.1 Performance and Limitations of the AHL Model

The Avrami-Hoffman–Lauritzen (AHL) theory, expressed by the combination of eqs 1–3, represents a standard depiction of the nucleation–growth behavior in polymer matrices. It was derived specifically for the crystallization from the molten state (as opposed to the crystal growth within a frozen-in amorphous polymer matrix). The technologically more common, as well as physico-chemically more important case is that of the nucleation and crystal growth occurring during continuous cooling—Section 2.1.1. will be dedicated to this case. The performance of the AHL theory during the isothermal experiments will be showcased in Section 2.1.2.

#### 2.1.1 AHL Under Nonisothermal Conditions

The present section aims to analyze the performance of the AHL model under nonisothermal (linear cooling) conditions. Based on eqs 1 and 2, the derivative AHL data (i.e., dα·d*t*^–1^, similar to what is measured by, e.g., DSC) will be simulated at three *q*^–^ (0.5, 5, and 50 °C·min^–1^, covering the most common range of technologically available conditions) for a variety of the materials’ kinetic parameters to explore their influence on the shape of the dα·d*t*^–1^-*T* peaks. In this regard, particular attention will be paid to the mutual positions of the dα·d*t*^–1^-*T* peaks and the inflection point of the onset edge of the *K*-*T* dependence (where *K* is expressed by eq 2). Note the recent study^49^ in which a direct relation was identified between the distortion of the Avrami kinetics and the manifestation of the crystallization process in the vicinity of the inflection point of the non-Arrhenian *K*-*T* dependence (the study was focused on the crystallization in glasses and glass-ceramics).

**Figure 2:**
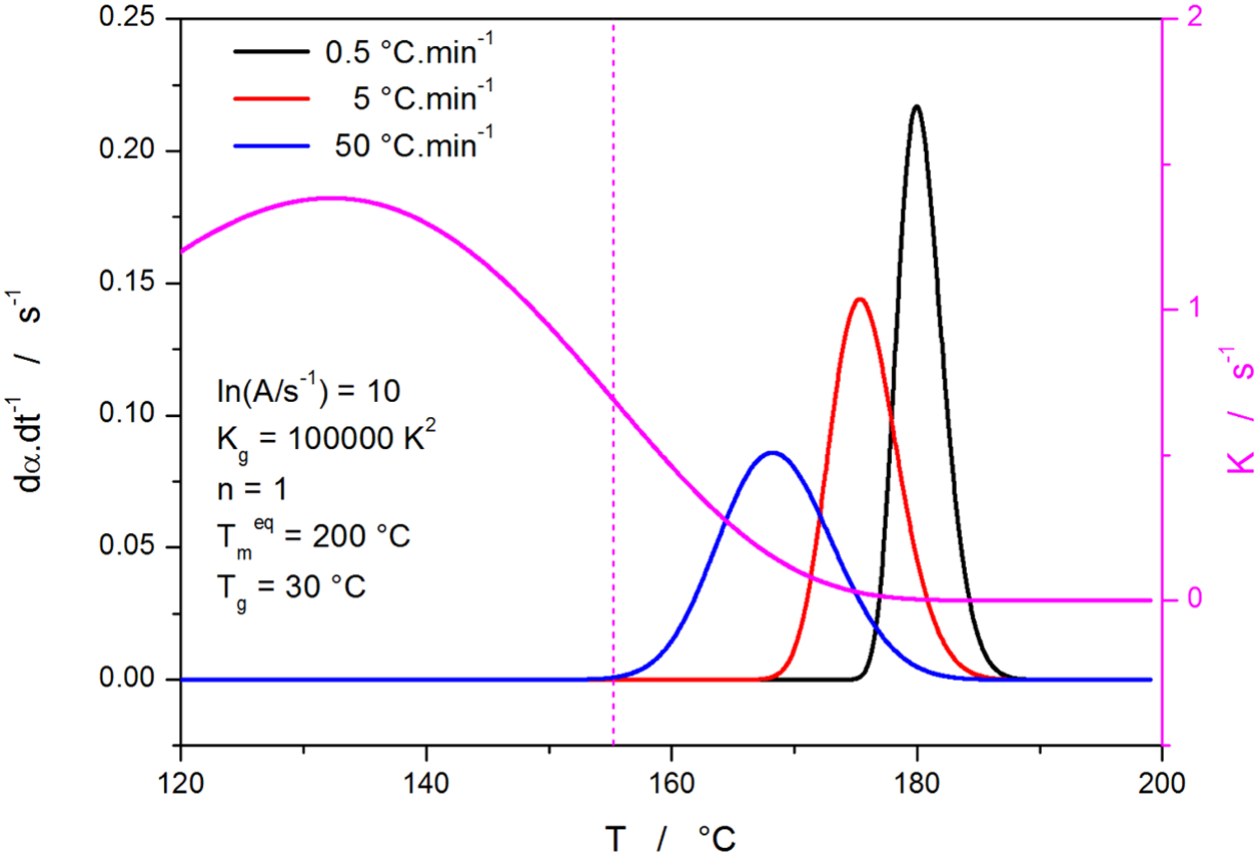
Baseline AHL data set (denoted ″A″ later, in Table 1) of the three crystallization kinetic peaks simulated for *q*^–^ = 0.5, 5, and 50 °C·min^–1^, and for the HL parameters listed in the graph. The magenta curve associated with the right-side axis depicts the temperature dependence of *K* (calculated according to eq 2); the vertical dotted line indicates the onset inflection point of this dependence.

**Figure 3:**
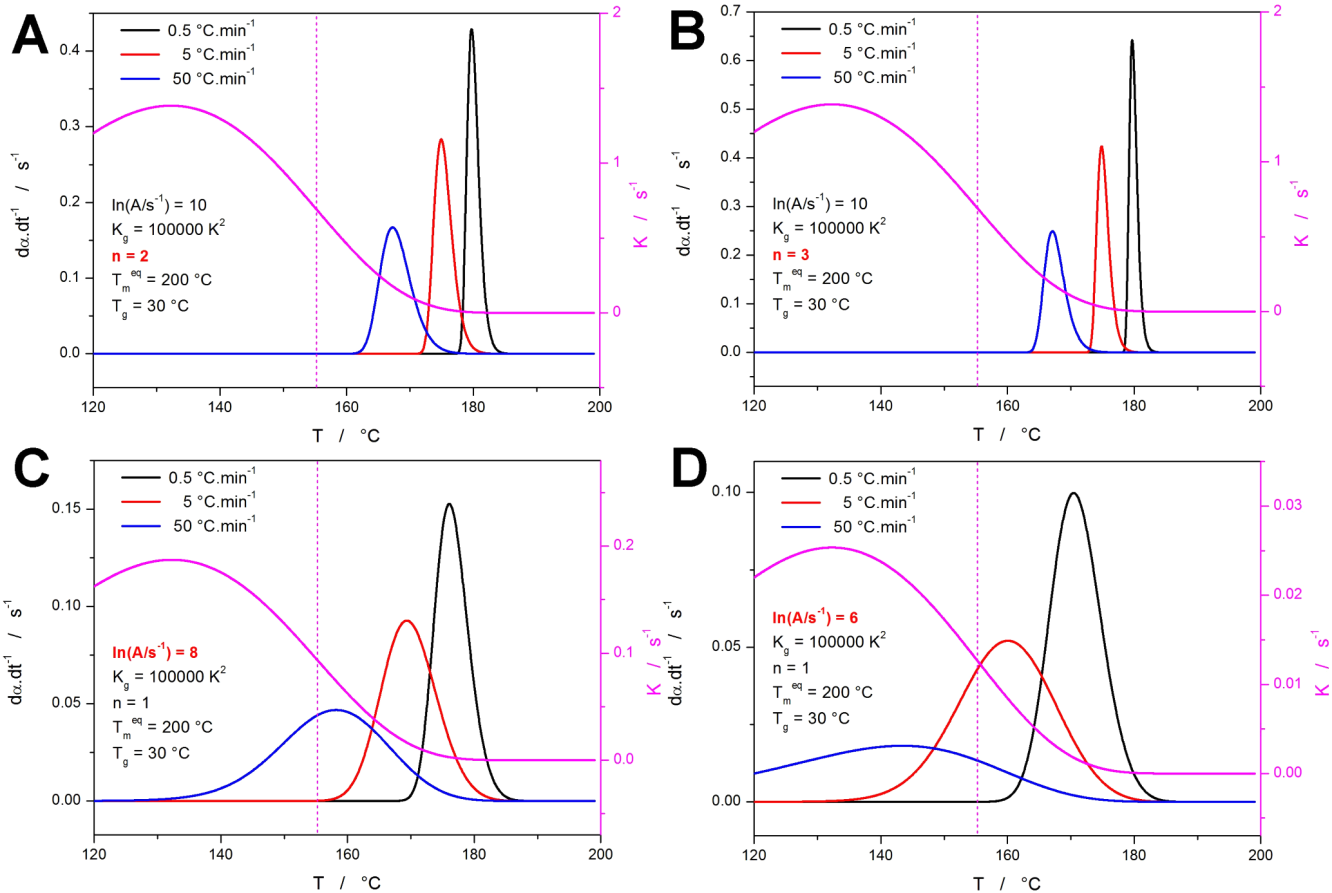
(a–d) Simulated data sets (denoted “B—E” in Table 1) demonstrating the influences of changing *n* and ln *A*. The magenta curve associated with the right-side axis depicts the *K*-*T* dependence; the vertical dotted lines indicate the onset inflection point of the *K*-*T* dependences.

In Figure 2, the (arbitrarily chosen) baseline data set is depicted, simulated for ln(*A*/s^–1^) = 10, *K*_g_ = 100,000 K^2^, *n* = 1, *T*_m_^eq^ = 200 °C, and *T*_g_ = 30 °C (resulting in *T*_∞_ = 0 °C). In this case, the crystallization proceeds fully at the initial concave onset of the *K*-*T* dependence, the borderline of which is given by the vertical dashed line indicating the *K*-*T* point of inflection. Correspondingly, the kinetic dα·d*t*^–1^-*T* peaks exhibit a slower onset and a more rapid endset, represented by skewing to lower *T*. The first set of variables, the effect of which was explored, is shown in Figure 3. With *n* increasing to 2 and 3 (Figure 3a,b, respectively), the crystal growth proceeds within “higher dimensions”, consuming the amorphous matrix faster. This results in higher and narrower kinetic peaks, shifting closer to their origin and further from the *K*-*T* inflection point—i.e., the typical Avrami asymmetry (skewing to higher α values) is preserved. The opposite effect is demonstrated in the case of the varying *A* in Figure 3c,d. The lower the *A*, the slower the crystallization process due to the decrease of *K* (*A* works as a simple multiplier within eq 2). Consequently, the kinetic peaks shift to a lower temperature (beyond the *K*-*T* inflection point), which leads to a further slowdown of the transformation of the remaining portion of the amorphous/noncrystalline matrix. The associated prolongation of the endset peak tail essentially results in the change of the peak asymmetry, skewing it to a higher temperature (lower α).

**Figure 4:**
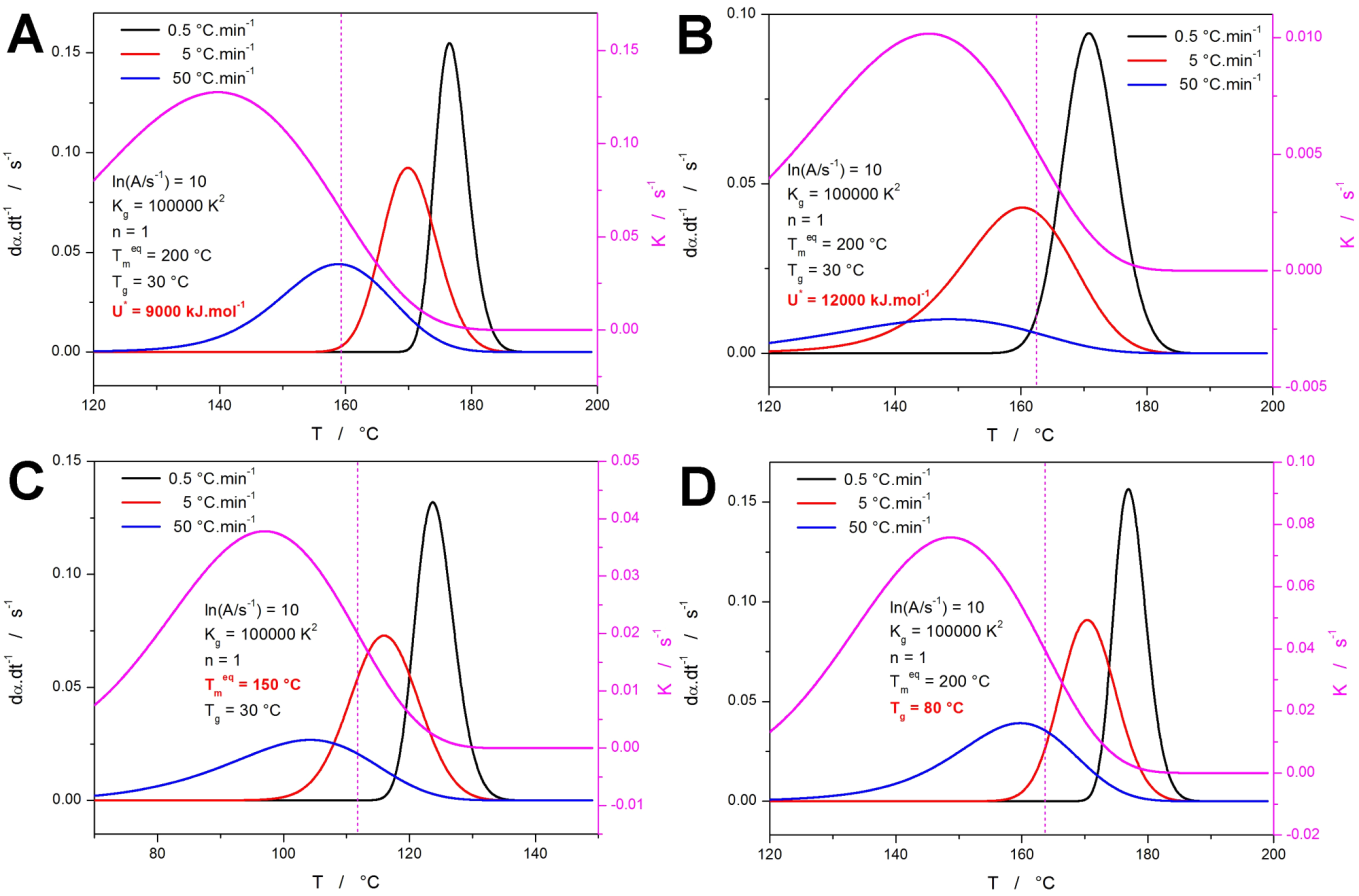
(a–d) Simulated data sets (denoted “F–I” in Table 1) demonstrating the influences of changing U*, *T*_m_^eq^, and *T*_g_. The magenta curve associated with the right-side axis depicts the *K*-*T* dependence; the vertical dotted lines indicate the onset inflection point of the *K*-*T* dependences.

The variables explored so far have not influenced the position of the *K*-*T* inflection point. This will not be the case from now on. In Figure 4, the influences of varying U*, *T*_m_^eq^, and *T*_g_ are shown. Despite being considered a constant of 6,300 J·mol^–1^, certain literature data indicate that U* can vary for different families of polymeric materials. By increasing the energy barrier U* (essentially representing a portion of the activation energy associated with the crystallization process), the *K*-*T* dependence decreases in absolute magnitude, and the width of its peak gets narrower—see Figure 4a,b. The corresponding decrease of the crystallization rate again leads to the slowdown of the crystallization process, shifting its course beyond the *K*-*T* point of inflection and changing the characteristic Avrami asymmetry. At the higher U* values, the crystal growth process is so slow that only partial crystallinity is reached (α not reaching 100%). A similarly large change can also be invoked by decreasing *T*_m_^eq^ (see Figure 4c), which results in the dα·d*t*^–1^-*T* and *K*-*T* dependences being shifted to lower temperatures and in the *K*-*T* dependence decreasing in both absolute magnitude and broadness. Akin effect can also be achieved by increasing *T*_g_, as is depicted in Figure 4d. In both these cases, the fundamental cause is represented by the increasing positive contribution to *K* from the undercooling (driving force of the crystal growth) being earlier outweighed (due to the smaller temperature gap between *T*_g_ and *T*_m_^eq^) by the negative contribution of the decreasing self-diffusion of the polymeric chains (associated with *T* continually decreasing toward *T*_g_ and *T*_∞_). The main point of these demonstrations is, concerning the present study, again the marked change of the asymmetry of the kinetic peaks beyond the *K*-*T* inflection point (i.e., at temperatures lower than the one corresponding to the *K*-*T* inflection point).

**Figure 5:**
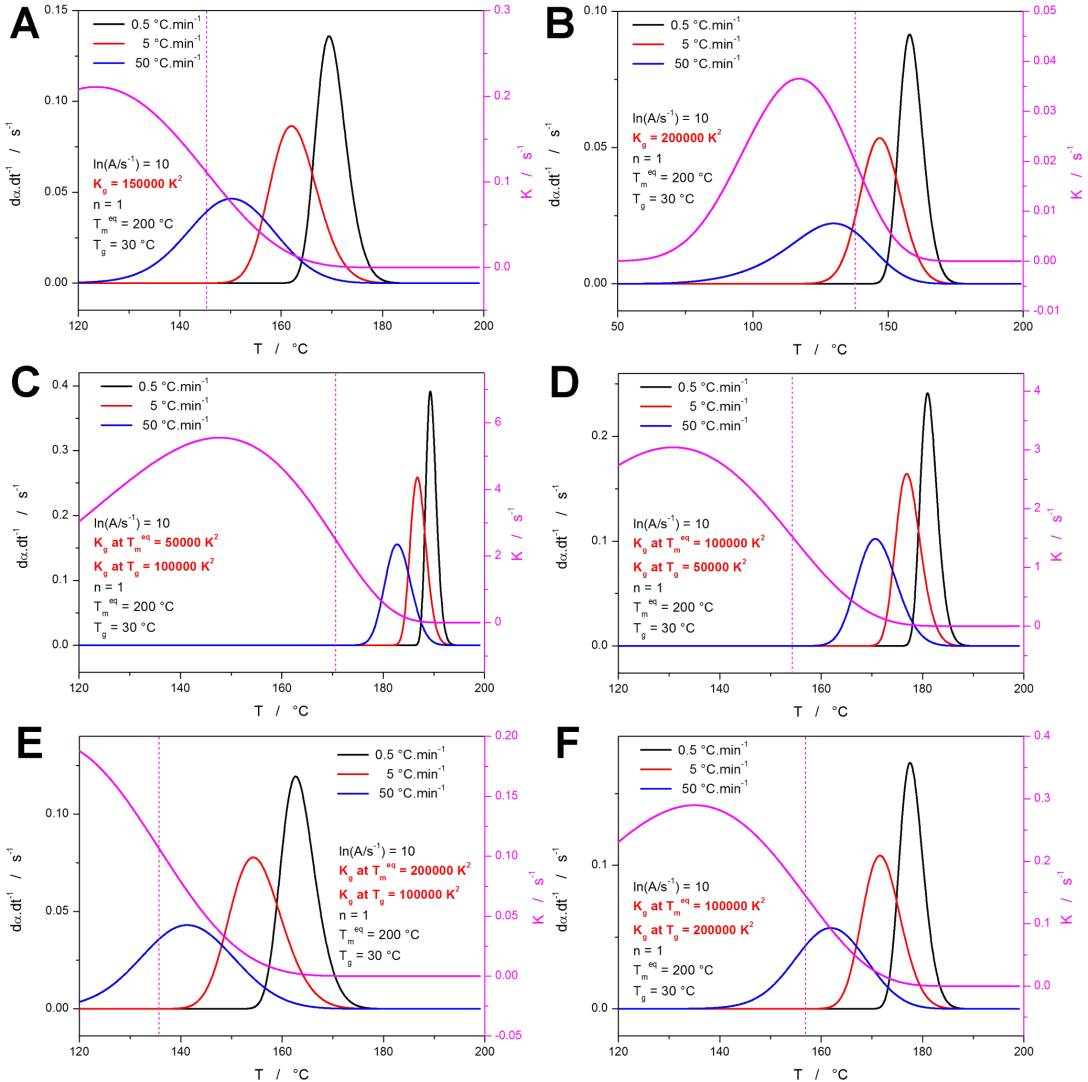
(a–f) Simulated data sets (denoted “J–O” in Table 1) demonstrating the influence of changing *K*_g_. The magenta curve associated with the right-side axis depicts the *K*-*T* dependence; the vertical dotted lines indicate the onset inflection point of the *K*-*T* dependences.

Particularly interesting are the simulations displayed in Figure 5 which introduce various temperature dependences of *K*_g_ into eq 2. Similarly to the quantities analyzed in Figure 4, *K*_g_ also influences the magnitude and position of the maximum of the *K*-*T* dependence. However, contrary to those quantities (U*, *T*_m_^eq^, *T*_g_), *K*_g_ can change with *T*, either through *w*_n_ (the nucleation regime constant) (eq 3), or via a more complex apparent homogeneous/heterogeneous mechanism, where the overall rate constant is partially compensated through the pre-exponential factor (*A*).^1,12−14^ Starting with the influence of the increasing (but temperature-wise constant) *K*_g_ value (shown in Figure 5a,b), the temperatures corresponding to the maxima and inflection points of the *K*-*T* dependence decrease, while the *K*-*T* peak gets slightly narrower: *T*_max_*^K-T^* = 132.3 °C and T_inf_*^K-T^* = 155.2 °C for *K*_g_ = 100,000 (Figure 2), *T*_max_*^K-T^* = 123.5 °C and *T*_inf_*^K-T^* = 145.3 °C for *K*_g_ = 150,000 (Figure 5a), *T*_max_*^K-T^* = 117.1 °C and *T*_inf_*^K-T^* = 137.9 °C for *K*_g_ = 200,000 (Figure 5b). The main influential effect is, however, associated with the increase of the apparent activation energy for the nucleation process (expressed by *K*_g_—see eq 2), and the consequent decrease of the absolute magnitude of *K*.

Similarly to the effect of U*, this again slows down the overall crystallization process so that at higher cooling rates, a significant portion of the crystal growth occurs beyond the *K*-*T* inflection point. For the *K*_g_-*T* dependences simulated as linearly increasing or decreasing within the *T*_g_-*T*_m_^eq^ temperature range (as suggested in the corresponding legends of Figure 5c–f), the following combinations of the rate constant characteristic temperatures were obtained: *T*_max_*^K-T^* = 147.8 °C and T_inf_*^K-T^* = 170.6 °C (Figure 5C), *T*_max_*^K-T^* = 130.7 °C and *T*_inf_*^K-T^* = 154.3 °C (Figure 5d), *T*_max_*^K-T^* = 114.3 °C and *T*_inf_*^K-T^* = 135.7 °C (Figure 5e), *T*_max_*^K-T^* = 135.1 °C and *T*_inf_*^K-T^* = 156.9 °C (Figure 5f). Since each of the implemented *K*_g_-*T* dependences either starts or finishes at 100,000 K^2^, it is convenient to compare the *T*_max_*^K-T^* and *T*_inf_*^K-T^* values with those obtained for constant *K*_g_ = 100,000 K^2^ (Figure 2; *T*_max_*^K-T^* = 132.3 °C and *T*_inf_*^K-T^* = 155.2 °C). Clearly, the characteristic *K*-*T* temperatures (and, correspondingly, the overall crystallization behavior) resemble the data for the *K*_g_ values that cover the relevant temperature region. For the present case, the (arbitrarily chosen) baseline behavior depicted in Figure 2 is best resembled by the *K*_g_-*T* dependences with the implemented *K*_g_ = 100,000 K^2^ at *T*_m_^eq^ (Figure 5d,f). It must be, however, stressed that the *K*_g_-*T* dependence still largely influences the absolute value of *K* (as implied from the comparison of, e.g., Figures 2 and 5f), which makes the potential identification of the nonconstant *K* highly relevant for the modeling of the nucleation/crystallization processes in polymers. The data sets simulated in this section are summarized in Table 1; for simplicity of further identification, they are identified by letters “A”–“O”.

**Table 1:** Parameters for the Non-Isothermal Cooling Simulations Performed in Terms of the AHL Model^a^

Simulation no.	Figure no.	ln (*A*/s^–1^)	*n* -	*T*_g_ °C	*T*_m_^eq^ °C	U* kJ·mol^–1^	*K*_g_ kK^2^
A	2	10	1	30	200	6300	100
B	3A	10	**2**	30	200	6300	100
C	3B	10	**3**	30	200	6300	100
D	3C	**8**	1	30	200	6300	100
E	3D	**6**	1	30	200	6300	100
F	4A	10	1	30	200	**9000**	100
G	4B	10	1	30	200	**12000**	100
H	4C	10	1	30	**150**	6300	100
I	4D	10	1	**80**	200	6300	100
J	5A	10	1	30	200	6300	**150**
K	5B	10	1	30	200	6300	**200**
L	5C	10	1	30	200	6300	100 → 50
M	5D	10	1	30	200	6300	50 → 100
N	5E	10	1	30	200	6300	200 → 100
O	5F	10	1	30	200	6300	100 → 200

a The sets are identified by the corresponding kinetic parameters (changes with respect to the baseline dataset a are in bold).

**Figure 6:**
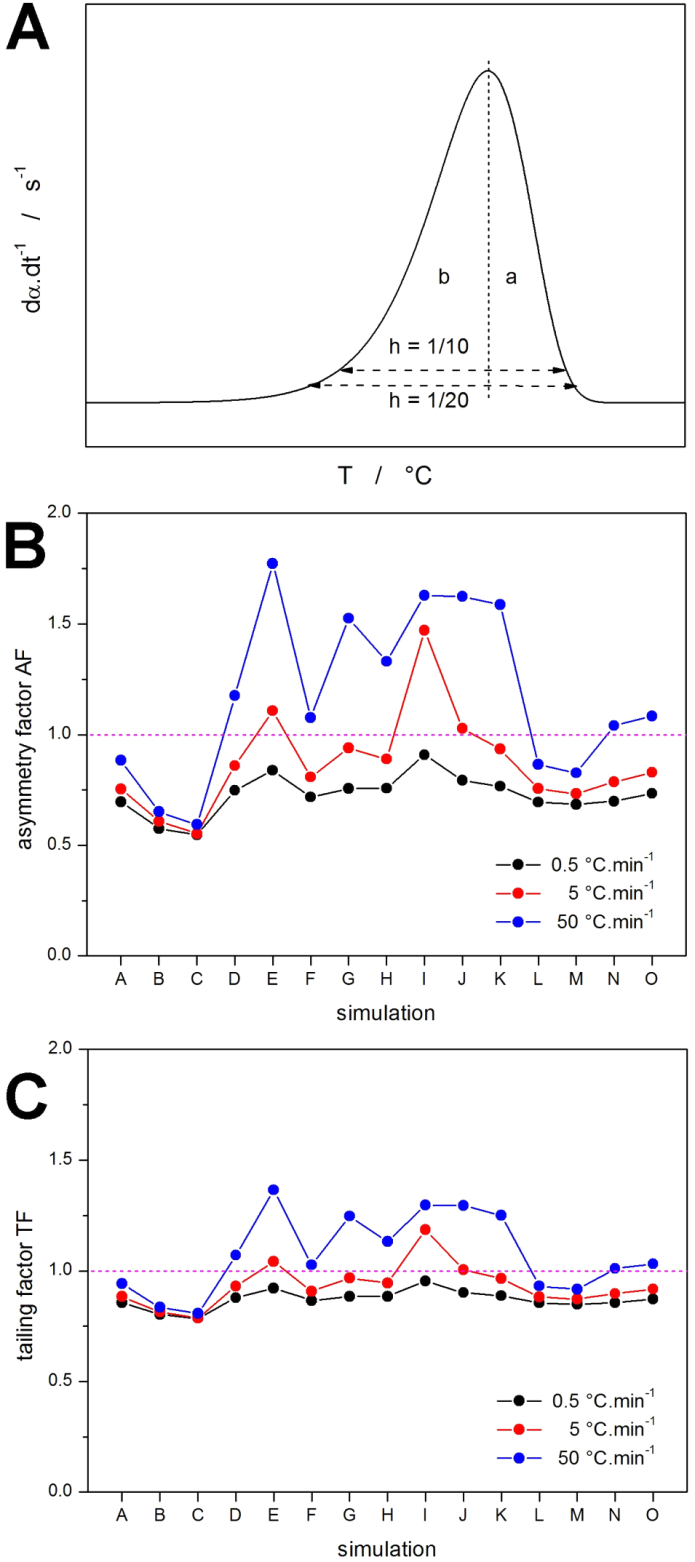
(a) Schematic determination of the distances utilized in eqs 5 and 6 for calculating AF and TF. (b,c) The AF and TF values calculated for the simulated crystallization peaks shown in Figures 2–5 (the individual data sets are identified by letters referring to Table 1).

To quantify the above-described changes concerning the main topic of the present paper, the asymmetry factor (AF) and tailing factor (TF) can be defined using eqs 5 and 6, respectively:
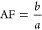
5
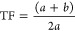
6where *a* and *b* are the distances in 1/10 (for AF) or 1/20 (for TF) of the peak height that indicate the corresponding peak half-widths (a schematic depiction of their determination is shown in Figure 6a). Note that typically, the peaks evolve from left to right (e.g., during heating with increasing temperature, or in spectroscopy with increasing wavelength on the X axis), where *a* corresponds to the initial (left onset edge) part of the peak, and *b* corresponds to the final (right endset edge) part of the peak. In the present case of crystallization at cooling, as the data are simulated/recorded on the *X*-axis from right to left, we have switched the definition of the *a* and *b* distances (see Figure 6a) so that the AF and TF values correctly denote the skewing to lower or higher values of α. In particular, AF (and TF) < 1 indicates the skewing to lower *T* and higher α, which is typical for the Avrami model. Correspondingly, the increase of AF and TF above 1 indicates the change in the AHL asymmetry, which is being discussed within the framework of the present paper.

The AF and TF data obtained from Figures 2**–**5 is shown in Figure 6b,c, respectively. The individual simulation sets are denoted by letters from Table 1. The kinetic peaks simulated in terms of the AHL model exhibit consistent evolvement of the peak tails, as indicated by a similar dependence (apart from the absolute scaling) of the AF and TF asymmetries on the input parameters of the simulations. Note that at AF = TF = 1, the peaks would be perfectly symmetric; the AF and TF values <1 indicate the asymmetry typical for the Avrami model, and the values >1 show the distortion due to the crystallization proceeding at less than the exponential *K*-*T* dependence (i.e., the data beyond the *K*-*T* inflection point). The major conclusion derived from the AF and TF data is that for the AHL model, these values always have a marked dependence on *q*^–^, with both factors significantly increasing with increasing *q*^–^. This is a key condition for the AHL-based description of any experimental data exhibiting the anti-Avrami asymmetry (i.e., the skewing to low α values). Based on the experimental observations (a few examples shown in Figure 1), this is often not the case, and the anti-Avrami asymmetry (or at least a skewed Avrami asymmetry) is more-or-less constant in magnitude and independent of *q*^–^.

**Figure 7:**
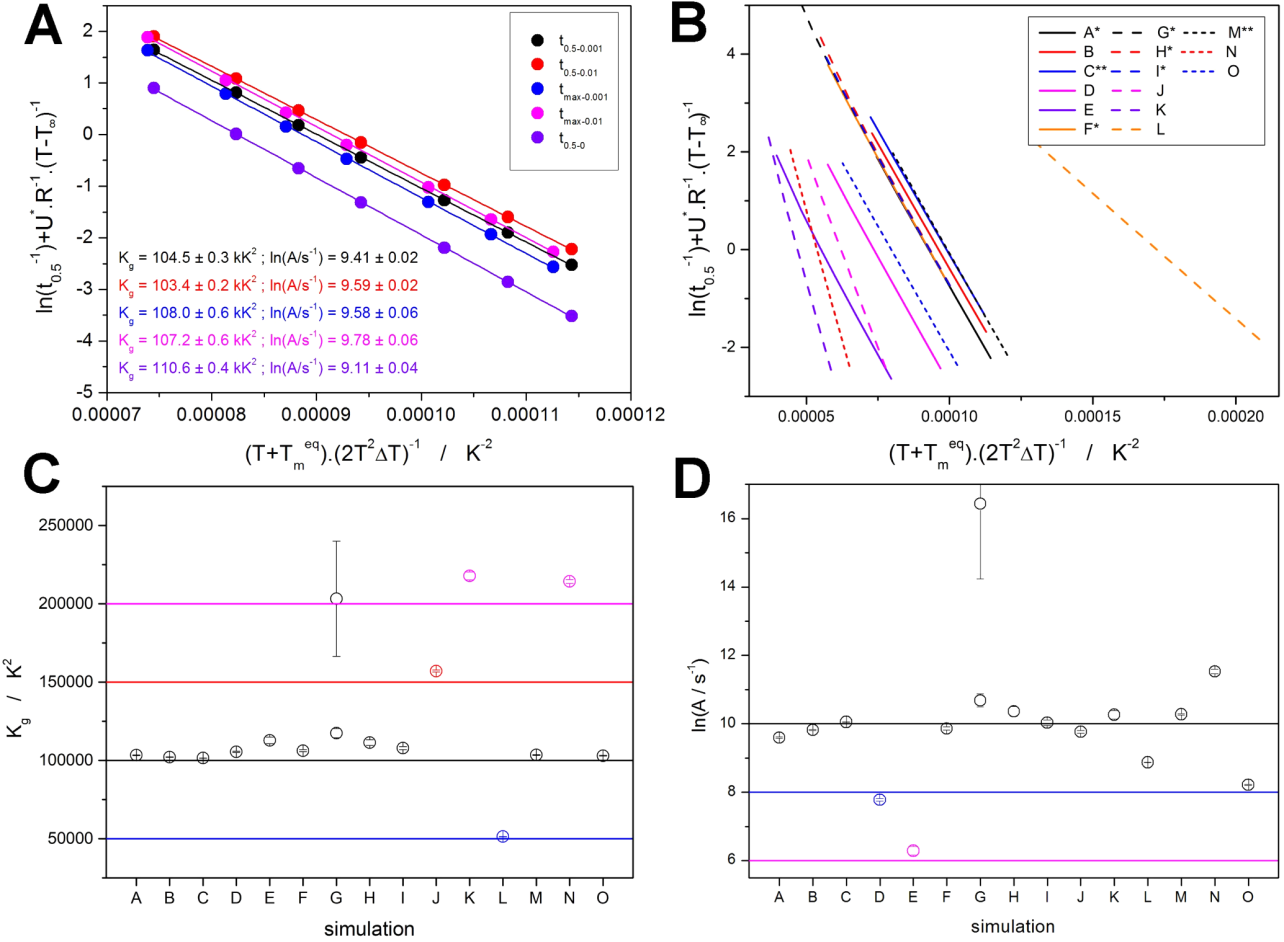
(a) The “*t*_0.5_ methodology” applied to data set A from Table 1, testing the impact of differently determined characteristic times. The subscripts indicate α values (scaled from 0 to 1) used to calculate the characteristic time used in eq 7 (“max” denotes α corresponding to the peak maximum). (b) The *t*_0.5–0.01_ dependences calculated for all data sets from Table 1. (c,d) *K*_g_ and ln *A* values determined according to eq 7 from the dependences depicted in (b). Horizontal lines and the coloring indicate the corresponding true values input into the simulations.

To gain further insight into the performance and behavior of the AHL kinetics in the cases of the *K*-*T* inflection point interaction with the standard crystallization kinetics, the crucial evaluation procedure associated with this model, namely the determination of *K*_g_ and ln *A* from the *t*_0.5_ values, was applied to the data sets A–O. The methodology can be expressed by eq 7:
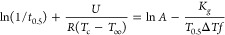
7where *t*_0.5_ and *T*_0.5_ are the time and the temperature associated with α = 50%. Equation 7 represents an independent linearization method for the determination of *K*_g_ and ln *A*, which can be consequently used as invariable inputs into the nonlinear optimizations fitting the experimental data with the AHL model. Since this evaluation is fundamental for reducing the number of variables during the AHL modeling/description of the experimental data, we have checked the functionality of this method when encountering the simulated *K*-*T*-based distortions of the crystallization peak (as described in the present section). Before the corresponding calculations were performed, an appropriate definition of *t*_0.5_ had to be chosen—note that in the case of the nonisothermal data, the induction period cannot be easily determined; thus, a variety of ways how to determine *t*_0.5_ exists. In Figure 7a, several ways of defining *t*_0.5_ are presented: the upper limit was chosen either to correspond exactly to α = 50% or to be identical with the maximum of the crystallization peak; the lower limit (essentially a correction for the induction period) was selected to be either 0.1, 1, or 0% (time zero identical with the initiation of cooling at *T*_m_^eq^). The resulting *K*_g_ and ln *A* values (see Figure 7a) show that the *t*_0.5_ calculated as the difference of times corresponding to α = 1% and α = 50% (in the following text denoted as *t*_0.5–0.01_) can be considered the most accurate option, with both quantities (*K*_g_ and ln *A*) being determined with less than 5% error. This definition of *t*_0.5_ was used in all consequent evaluations.

The *t*_0.5–0.01_ dependences obtained for the data sets simulated within the framework of Section 2.1.1. are shown in Figure 7b—see Table 1 for the interpretation of the individual simulation parameters. Most of these dependencies are linear (the only significant exceptions were data sets E and G), indicating that even the large distortions of the AHL asymmetry (data sets C–K) do not necessarily impair the utilization of the discussed methodology. This is even more evident from Figure 7c,d, where the resulting *K*_g_ and ln *A* values (determined for the individual data sets from dependences depicted in Figure 7b) are compared with the true values (horizontal dashed lines) input into the simulations. The only exception was data set G being largely curved at the highest simulated cooling rates, which resulted in a major deviation from the true value—see the caption of Figure 7c,d for details; the full unzoomed Figure 7b is shown in the Supporting Information material. Otherwise, both *K*_g_ and ln *A* can be determined reasonably accurately despite the distortions of the kinetic peaks caused by the involvement of the change of the *K*-*T* curvature beyond the inflection point.

#### 2.1.2 AHL Under Isothermal Conditions

Whereas the polymer crystallization during the cooling of the melt is unambiguously defined by the material’s thermal history, the isothermal annealing can, in theory, proceed either after cooling of the melt or after heating of a frozen-in amorphous/glassy state. The former option is often relevant for relatively rapidly crystallizing polymers, which cannot be reasonably easily prepared amorphous in bulk amounts. In such a case, *K* would be rather high, and an isothermal annealing experiment at temperatures below *T*_inf_*^K^*^–*T*^ would be difficult to realize. On the other hand, the slowly crystallizing polymers with a low absolute magnitude of *K* can be prepared amorphous, and achieving virtually any temperature for the isothermal crystallization experiment is generally possible. Nonetheless, despite these common experimental limitations, in the present section, the theoretical simulations will be performed for all archetypal combinations of the absolute magnitude of *K* and mutual positions of *T*_inf_*^K^*^–*T*^ and the constant annealing temperature (*T*_a_).

**Figure 8:**
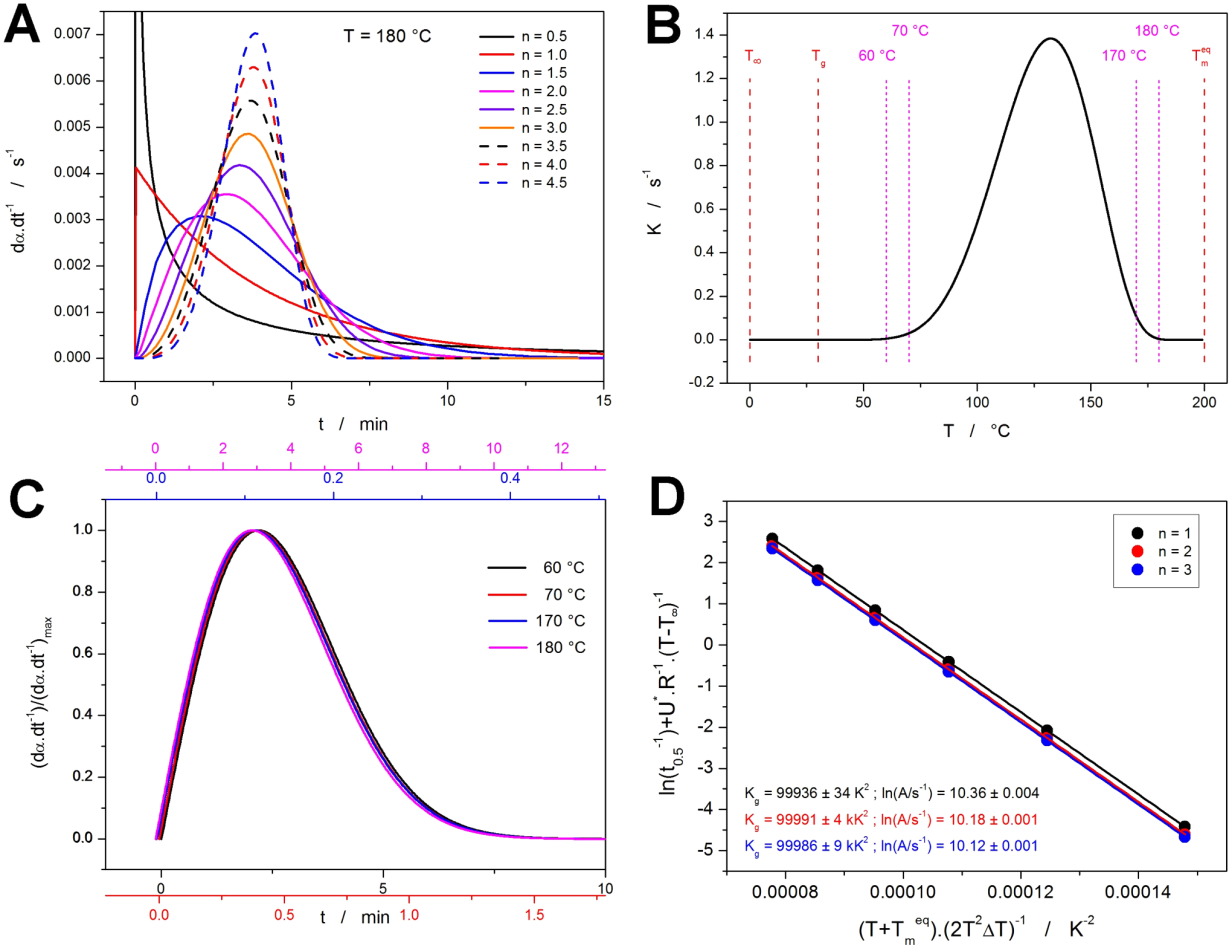
(a) The AHL kinetic peaks simulated for a variety of *n* and isothermal annealing at 180 °C (the rest of the kinetic parameters were identical to those of data set A from Table 1). (b) The temperature dependence of *K* corresponding to data set A from Table 1. The dashed vertical lines indicate *T*_∞_, *T*_g_, and *T*_m_^eq^; the dotted vertical lines indicate the four selected isothermal annealing temperatures. (c) Isothermal AHL kinetic peaks (normalized to their height) simulated for *n* = 2, temperatures, and *K* parameters indicated in (b). The coloring of the individual peaks corresponds to the respective X axes. The peaks are very purposefully slightly shifted along the *X*-axis so that all colors are visible (otherwise, perfect overlaps would be achieved). (d) Test of the *t*_0.5_ methodology (eq 7) for the isothermal data simulated for different *n* values, kinetic parameters corresponding to set A from Table 1, and temperatures ranging between 170 and 185 °C.

Figure 8a shows the typical asymmetries of the isothermal Avrami kinetics for the typically encountered range of *n*. Note that *n* reflects the type of nucleation, the growth dimension, and the rate-controlling mechanism.^13,14^ The series of examples from Figure 8a was calculated for the base set A of the kinetic parameters from Section 2.1.1. (ln(*A*/s^–1^) = 10, *K*_g_ = 100,000 K^2^, *T*_m_^eq^ = 200 °C, *T*_g_ = 30 °C, U* = 6,300 kJ·mol^–1^—see Table 1) and the isothermal annealing at *T*_a_ = 180 °C. Since all these quantities are either material constants or are only *T*-dependent and not time-dependent, *K* from eq 1 is a true constant under isothermal conditions. This means that only the dα·d*t*^–1^ and *t*_0.5_ magnitudes are influenced by the value of *K*, but the asymmetry of the given kinetics remains uninfluenced by the actual course of the *K*-*T* dependence. Note that the induction time (*t*_ind_) which can be sometimes incorporated into eq 1 as “(*t* – *t*_ind_)*^n^*” also does not influence the peak asymmetry. Hence, similarly to the Arrhenian concept, also the Hoffman–Lauritzen expression of *K* leads to a uniform set of the asymmetries of the crystallization peak (as depicted in Figure 8a). This is demonstrated in Figure 8b,c. For the *K*-*T* dependence corresponding to set A from Table 1, four temperatures *T*_a_ (indicated by the vertical magenta dashed lines in Figure 8b) were selected at the onset as well as at the end set of the *K*-*T* peak simulated for the above-mentioned kinetic parameters. For these temperatures, the isothermal dα·d*t*^–1^ signals were simulated for *n* = 2; the signals were then normalized to their absolute magnitude and plotted using individual scalings of the *X*-axis so that the best possible normalization of the peak width was achieved. At that time, the signals perfectly overlapped. Thus, an arbitrary slight shift along the *X*-axis was used for each signal so that the individual curves could be recognized in Figure 8c. Similar perfect overlaps are obtained for any other *n* and *K*-related kinetic parameters, assuming that only *T*_a_ is the variable in the given set of simulations. This demonstrates that for the isothermal measurements, the crystallization peak asymmetry is highly restricted, dictated solely by the value of *n* (in other words, the asymmetry cannot be changed/manipulated by optimizing other kinetic parameters included in eqs 1 and 2).

Similarly to the nonisothermal AHL simulations, the functionality of determining *K*_g_ and ln *A* from the *t*_0.5_ values was also verified for the case of isothermal annealing programs. Three sets of simulations were performed for the *n* = 1, 2, and 3; the other kinetic parameters were the same as listed above, and the isothermal annealing temperatures simulated within each set were *T*_a_ = 170, 173, 176, 179, 182, and 185 °C. The corresponding *t*_0.5_ dependences and the determined *K*_g_ and ln *A* values are shown in Figure 8d. As expected, both parameters are determined with a relatively high level of accuracy, with the ln *A* value being influenced by the initial α value used in the simulations (as described in detail in refs^50,51^).

### 2.2 Performance and Limitations of the ZCHL and MCHL Catalytic Models

As was shown in Section 2.1, the Avrami-Hoffman–Lauritzen kinetics is largely restricted concerning its ability to describe the experimentally occurring variety of the DSC crystallization peaks’ asymmetries. For this reason, the present paper introduces an in-depth analysis of two new and highly flexible solid-state kinetic models based on the Hoffman–Lauritzen nucleation–growth concept coupled with autocatalytic kinetics. In particular, the flexibility of the two new models is fundamentally implemented via the *N*^th^-order reaction kinetics (*N* is the reaction order), where the autocatalytic behavior (expressed through one additional parameter) further modifies the shape of the kinetic peak (offering either autocatalytic or autoretarding transformation mechanisms). Since both kinetic components are α-dependent, the models do not have an integral analytic solution (akin to eq 1 for the Avrami model) and must be expressed in their derivative form (eqs 8–10):
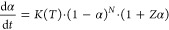
8
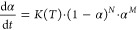
9
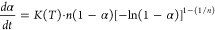
10where (1 – α)*^N^* is the standard *N*^th^-order reaction model, and the α*^M^* and (1 + *Zα*) terms represent two types of autocatalytic behavior (as analyzed for the Arrhenian rate constant kinetics, e.g., in refs^52−54^; eq 10 depicts the derivative form of the Avrami model for comparison. The variables *N*, *M*, and *Z* are the models’ parameters, which are formally considered to be material constants but are often temperature-dependent. Regarding their interpretation, *N* can be perceived as the reaction order from the *N*^th^-order reaction model. *M* and *Z* are the different expressions for autocatalysis, i.e., the increased crystallization rate with the increasing degree of conversion α. Whereas *Z* represents a true autocatalytic behavior, where the increase of this parameter additively increases the crystallization rate, the effect of *M* is more complicated. Since *M* is in the exponent, its effect leads to the decrease of the crystallization rate at low α, and then to the increase of the crystallization rate (more rapid evolution of the transformation pace at high α). Thus, the crystallization is delayed (the crystallization peak is shifted to lower *T* during cooling), but then it proceeds at a greater rate, producing a narrower and higher kinetic signal. In the present paper, the two models will be denoted as ZCHL (eq 8) and MCHL (eq 9). In compliance with the previous section, the present section will also contain two subsections dealing with the nonisothermal data (Section 2.2.1.) and isothermal data (Section 2.2.2.).

#### 2.2.1 ZCHL and MCHL Under Nonisothermal Conditions

**Figure 9:**
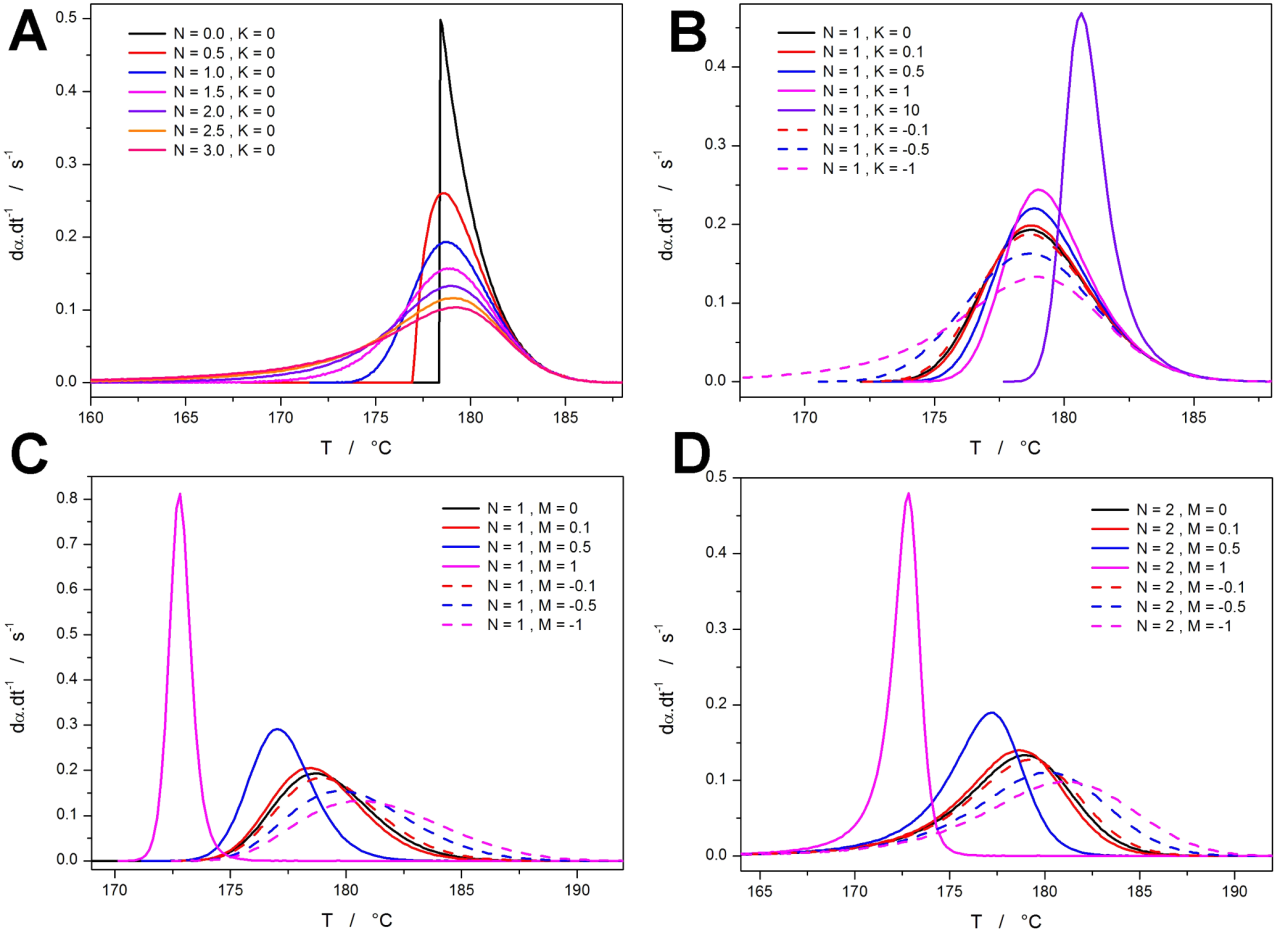
(a–d) Different nonisothermal ZCHL and MCHL kinetic peaks simulated for the HL parameters of set A from Table 1 and *q*^–^ = 1 °C·min^–1^.

The flexibility of the base *N*^th^-order reaction model (with either *M* or *Z* being equal to 0) for the nonisothermal cooling data is demonstrated (simulated) in Figure 9a. This kinetic component solves the main issue of the Avrami kinetics, i.e., the strict peak asymmetry associated with the model AHL, where the only changes of this asymmetry are introduced through the interaction with *K* (the discussion related to *T*_inf_*^K-T^* in Section 2.1.1). The range of asymmetries provided by the *N*^th^-order model kinetics fully covers all types of experimentally encounterable cases. An identical range of asymmetries is also obtained for any other *K*-*T* dependencies defined by the Hoffman–Lauritzen parameters. In the present paper, the *N*^th^-order kinetics is further modified by the individual autocatalytic terms. The influence of the (1 + *Zα*) term applied within the framework of the ZCHL model is demonstrated in Figure 9b—positive *Z* values lead to autocatalysis, and negative *Z* values result in autoretardation. Note that the autocatalysis can be imagined, e.g., as the crystal growth initiating from the secondary nucleation, while the autoretardation can occur as a consequence of the steric restrictions occurring at higher crystalline contents. The value of the *Z* variable can further markedly change the asymmetry of the kinetic peak, even transforming the original Avrami-like asymmetry to the anti-Avrami asymmetry (as shown in Figure 9b at increasingly negative *Z* values). Importantly, the autocatalytic term in the ZCHL model does not change the initial onset edge of the kinetic peak, which separates the influences of *Z* and the pre-exponential factor (*A*), making the physicochemical interpretation of the *Z* value more straightforward. A very similar impact of the *Z* value can also be observed for higher *N* values (see the Supporting Information material). An exemplary series of kinetic peaks simulated for the MCHL model is shown in Figure 9c,d. Contrary to the ZCHL model, the asymmetry given by the *N*^th^-order kinetic component changes only slightly by the *M* value, but the onset of the kinetic peak shifts significantly. As indicated by the data from Figure 9c,d, at increasingly positive *M* values, the kinetic peak is larger in magnitude. Still, it occurs at lower temperatures (the overall kinetics expressed by the analogy of *K* decelerates, but the process itself progresses more rapidly within a shorter time). At increasingly negative *M* values, the crystallization occurs earlier (at higher temperatures), but the process is slower and drawn out (the overall kinetics accelerates, but at the cost of the process progressing over a longer time). This consequence of the autocatalysis being expressed by the power function is somewhat counterintuitive and represents the main difference from the conceptually simpler ZCHL model.

**Figure 10:**
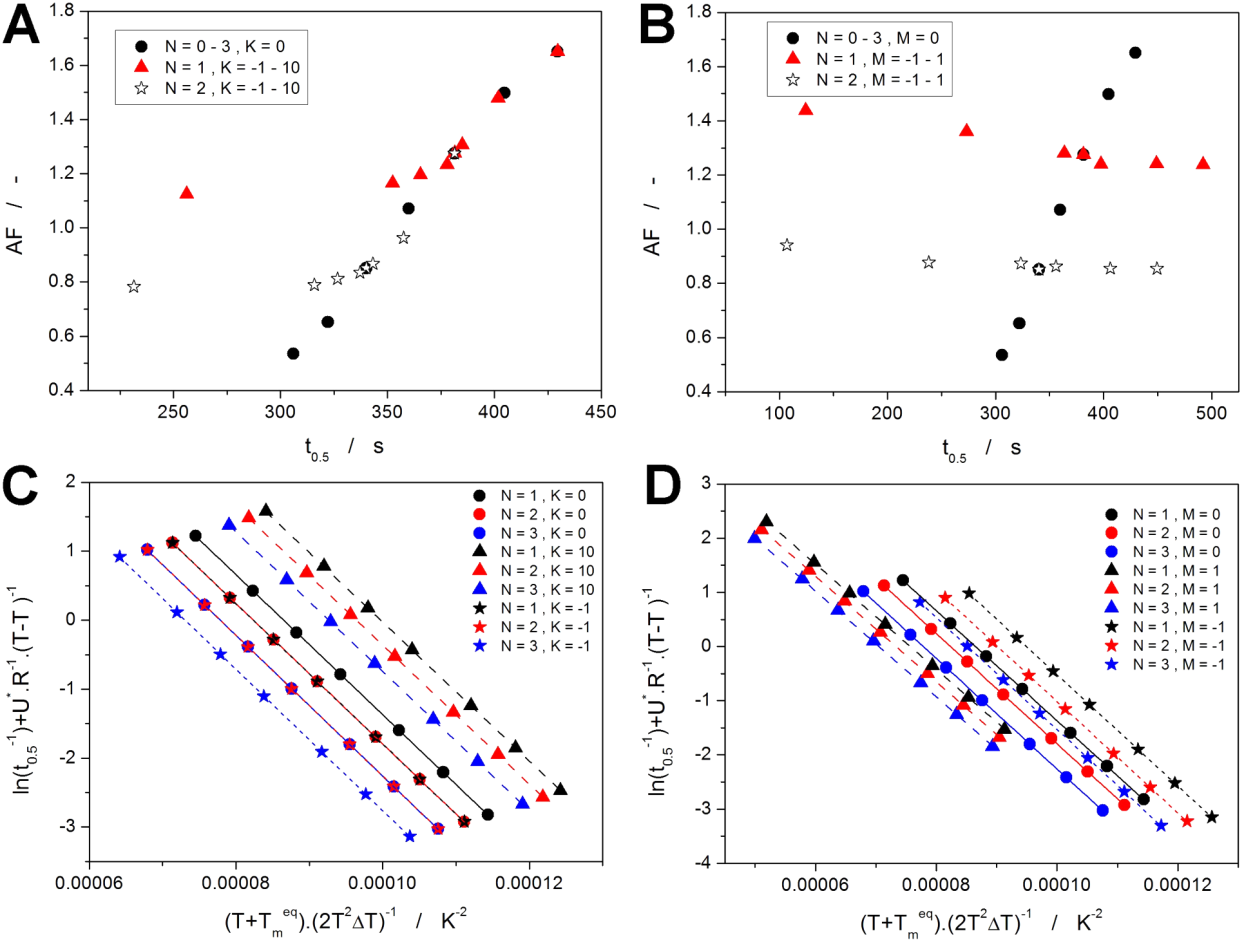
(a) AF indicators for the kinetic peaks depicted in Figure 9a,b (+ peaks similar to those from Figure 9b but simulated with *N* = 2). (b) AF indicators for the kinetic peaks depicted in Figure 9a,c, and d. (c) The *t*_0.5_ dependences simulated for the selected ZCHL peaks (autocatalytic parameters indicated in the legend, HL parameters corresponding to the data set A from Table 1, *q*^–^ = 0.5, 1, 2, 5, 10, 20, and 50 °C·min^–1^). (d) The *t*_0.5_ dependences simulated for the selected MCHL peaks (autocatalytic parameters indicated in the legend, HL parameters corresponding to the data set A from Table 1, *q*^–^ = 0.5, 1, 2, 5, 10, 20, and 50 °C·min^–1^).

A quantification of the nonisothermal ZCHL and AVHL behaviors is shown in Figure 10. In Figure 10a,b, the correlations between the peak asymmetry (expressed by AF from eq 5) and peak width (expressed by the *t*_0.5–0.01_; see Figure 7a) are shown for the two autocatalytic models. The ZCHL model influences the asymmetry/width ratio only at positive *Z* values (the dependences deviate from the base “*N* = 0–3, *Z* = 0” behavior), and for the negative *Z* values, the asymmetry/width ratio caused by the decrease of *Z* imitates the effect of increasing *N* within the *N*^th^-order kinetics term, i.e., (1 – α)*^N^*. On the other hand, the MCHL model very clearly distinguishes between the contributions of both terms, where the kinetic exponent *M* (in both positive and negative ranges of values) only changes the width of the kinetic peak and influences the peak asymmetry only minimally; the asymmetry is thus practically full dictated by the *N*^th^-order kinetics (parameter *N*). In addition to the shape-based quantification, the functionality of the *t*_0.5_-based methodology for the determination of *K*_g_ and ln *A* (see eq 7) was also tested for the data simulated within the framework of the ZCHL and MCHL models. The corresponding dependences (depicted in Figure 10c,d) are perfectly linear, predicting the *K*_g_ values with ± 2% errors for the ZCHL model and ± 3% errors for the MCHL model. Note that the ln *A* predictions largely deviated to lower values for both models, leaving this parameter to be included in the nonlinear optimization during the practical kinetic analysis of experimental crystallization data.

#### 2.2.2 ZCHL and MCHL Under Isothermal Conditions

**Figure 11:**
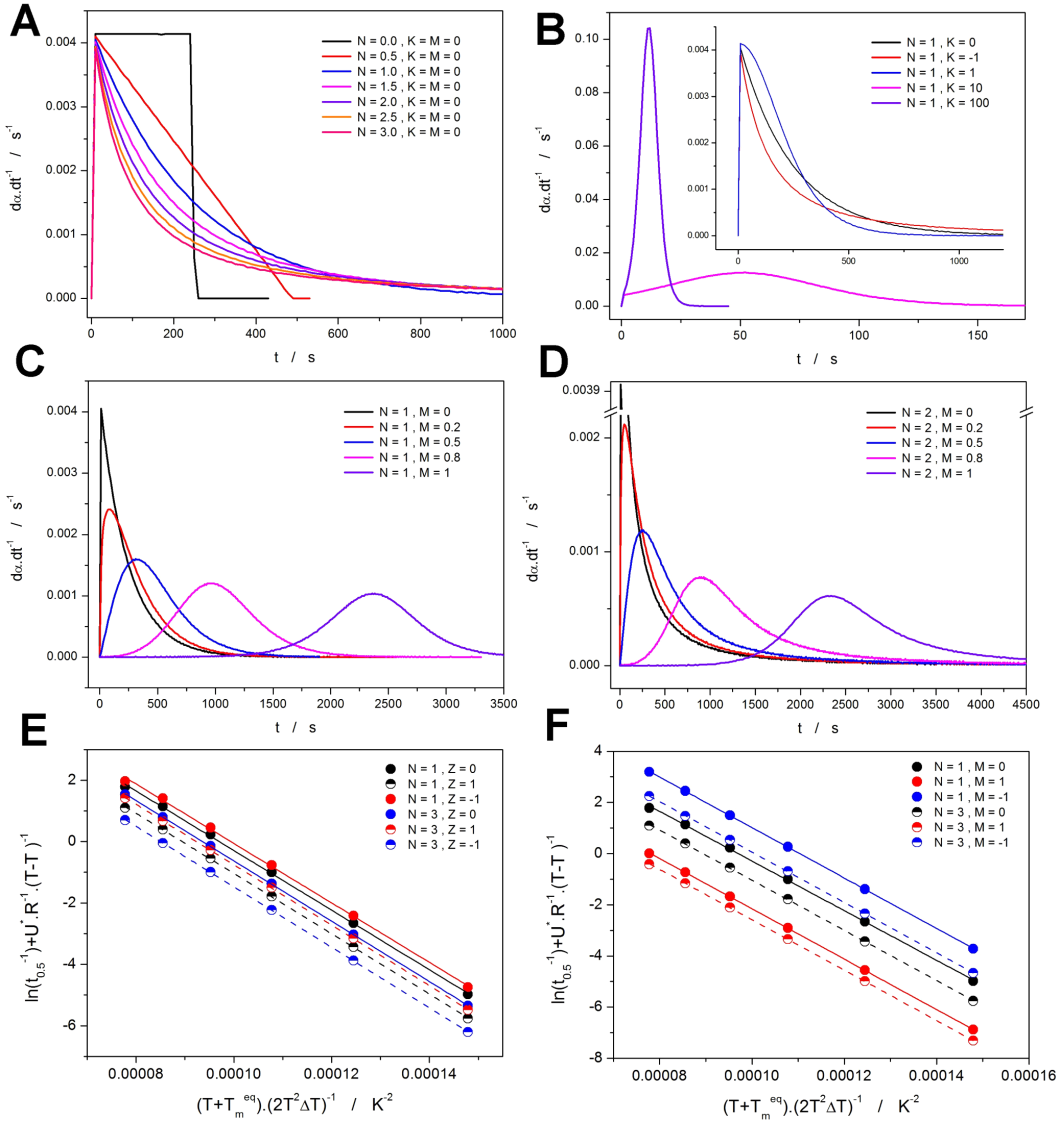
(a–d) Different isothermal ZCHL and MCHL kinetic peaks simulated for the HL parameters of set A from Table 1, and *T* = 180 °C. (e,f) The *t*_0.5_ dependences simulated for the selected ZCHL and MCHL peaks (autocatalytic parameters indicated in the legend, HL parameters corresponding to the data set A from Table 1, Ta = 170, 173, 176, 179, 182, and 185 °C).

Similarly to the nonisothermal analyses introduced in the previous section, also the assessment of the isothermal performance of the ZCHL and MCHL models will start with the introduction of the shape of the modeled kinetic peaks and with the influence of the individual model parameters (*M*, *N*, *Z*) on this shape. In Figure 11a, the basic shapes of the simulated isothermal crystallization peaks are shown for the sole *N*^th^-order kinetics (i.e., *Z* or *M* equal to 0). Since these peaks cover only a specific narrow group of the possible peak shapes (the variety of the AHL peaks in Figure 8a should be considered a mandatory minimum for the ZCHL and MCHL models that aspire to be “more flexible”), it is the role of the autocatalytic term to introduce the required shape variety. As is apparent from Figure 11b, the (1 + *Zα*) term from the ZCHL fails to meet this requirement, as the transition from the *N*^th^-order kinetics to the Avrami-like asymmetry is too slow and happens only in association with the shift of the onset edge to higher α. This limitation results in an inability of the ZCHL model to accurately describe even the standard isothermal Avrami kinetics, as was further verified using nonlinear optimization, where a series of Avrami isothermal peaks was attempted to fit by the ZCHL model (see the Supporting Information material for details). This result alone disqualifies the ZCHL model from any serious consideration of being used for modeling the crystallization in polymers.

Contrary to the ZCHL model, the MCHL model appears to cover a large variety of the shapes of kinetic peaks, including the reproduction of the Avrami-like kinetics—see Figure 11c,d. Whereas the increasing catalytic exponent *M* seemingly imitates the functionality of the Avrami exponent *n* from eq 1, the *N*^th^-order reaction exponent *N* is responsible for the skewing of the kinetic peaks without interfering in the course of the peaks’ onset (including the apparent imitation of the induction period). The ability of the MCHL model to simulate the Avrami kinetics was verified using nonlinear optimization, where various isothermal AHL peaks were fit by the MCHL kinetics—see the Supporting Information material for details.

Similarly to the nonisothermal tests introduced at the end of Section 2.2.1, the isothermal ZCHL and MCHL kinetics were also examined concerning the functionality of the *t*_0.5_-based methodology for estimating the *K*_g_ and ln *A* quantities. The results, practically identical to the nonisothermal ones, have shown that *K*_g_ can be estimated with a high level of precision (errors of ± 3% for both ZCHL and MCHL); the ln *A* quantity has again shown rather large deviations from what was input into the simulations.

### 2.3 Practical Utilization of the Autocatalytic Hoffman–Lauritzen Kinetics

It was shown in Section 2.2. that, out of the two tested autocatalytic Hoffman Lauritzen models, only the MCHL approach has high enough flexibility to imitate the standard Avrami kinetics, as well as to reproduce different potential skewings of the Avrami crystallization peaks (due to a variety of reasons). The ultimate flexibility of the MCHL model allows for a very accurate description of the experimental data, which in turn gives appropriate credit to any conclusions derived based on the interpretation of the mathematical description.

**Figure 12:**
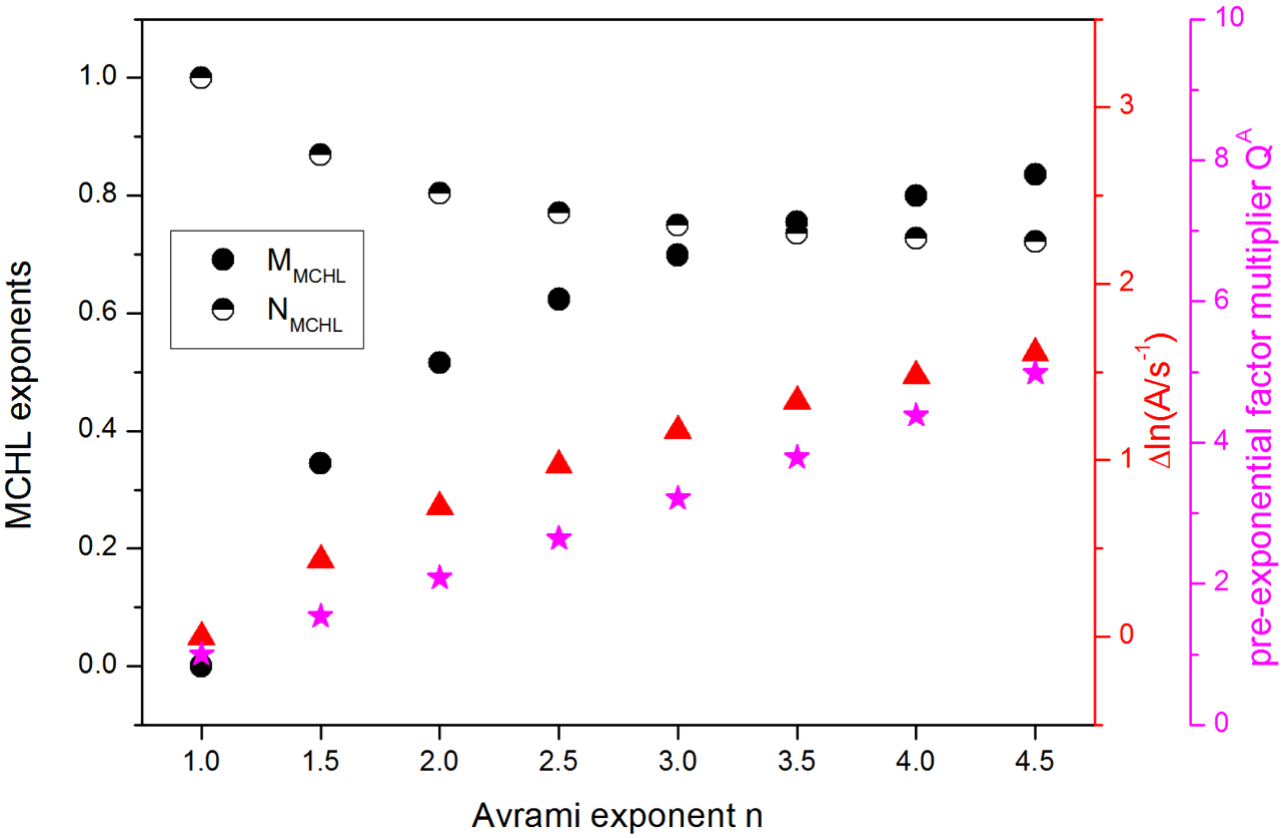
Correlations between the Avrami kinetic exponent (*n*) and the parameters of the MCHL model; Δln *A* and *Q*^*A*^ are defined by eqs 11 and 12, respectively.

In practice, the MCHL description of the polymer crystallization employing the MCHL model can be utilized in two ways. First, the potential deviation from the Avrami behavior can be quantified and interpreted in terms of the autocatalytic/autoretarding influence of, e.g., secondary nucleation and crystal growth. Thus, the relationship between the AHL and MCHL models must be established. Since no straightforward equality of the kinetic exponents arises from the comparison of eqs 8 and 9, we have simulated a large variety of the AHL kinetic peaks and used nonlinear optimization to fit them by the MCHL model (combination of eqs 2 and 8). The AHL kinetic peaks were simulated under both isothermal and nonisothermal conditions for different *K*-*T* dependences, as well as for different mutual positions of the kinetic peak and the *K*(T) inflection point so that all types of the AHL behavior described in Section 2.1. were covered, reproduced, and fit by the MCHL model. From these fits, a universal relationship between the AHL and MCHL models was established—as depicted in Figure 12. For each value of *n*, a unique MCHL description can be attributed, consisting of the specific values of the exponents *M* and *N*, and the (also exactly defined) change of *A* (eq 11):
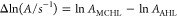
11

Note that the change of *A* is associated with the shift of the kinetic peaks to a lower temperature during cooling (as discussed in detail in Section 2.2.1, Figures 9C,D). Alternatively, the change of *A* can also be expressed in the form of the pre-exponential factor multiplier (*Q*^*A*^) (eq 12):
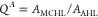
12which has an almost linear dependence on *n*—see Figure 9.

The dependences depicted in Figure 12 were further fit by polynomial functions to establish the accurate links between the monitored MCHL parameters and *n*. The relationship between the MCHL and AHL models is very rigorous—the standard errors arising from averaging all MCHL fits of the AHL peaks simulated under different conditions are more than 2 orders of magnitude smaller than the magnitude of points in Figure 12. Therefore, the higher orders of the polynomial fits were used, so that the corresponding correlation coefficients were *R*^2^ > 0.999999. For each MCHL parameter, a version of the following equations was calculated (eq 13):
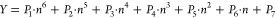
13where *P*_1_–*P*_6_ are numerical constants, *n* is the Avrami kinetic exponent, and *Y* is the particular MCHL quantity (*M*, *N*, Δln *A*, *Q*^*A*^). The numerical constants for each MCHL quantity are listed in Table 2. In this way, any description of the polymer crystallization data quantified in terms of the MCHL model can be related to the potential deviations from the standard Avrami behavior and consequently interpreted in terms of the specific type of its shape/asymmetry change. An increase/decrease of *A* (with *M* and *N* corresponding to the expected Avrami kinetics) may suggest a respective decrease/increase in the induction period. An increase/decrease of the kinetic exponent *M* may indicate a respective autoretardation/autocatalysis associated with the combined effects of the transition between the diffusion and interface-controlled growth and secondary nucleation/growth. An increase/decrease of the kinetic exponent *N* may then be associated with the effects of nucleation and growth acceleration/inhibition due to the steric restrictions and/or internal viscosity changes induced by factors other than the temperature.

**Table 2:** Polynomial Fit of the Dependences Shown in Figure 12 Following eq 13

*Y*	*P*_1_	*P*_2_	*P*_3_	*P*_4_	*P*_5_	*P*_6_	*P*_7_
*M*	–0.00239644	0.04530508	–0.35417078	1.47753138	–3.53394168	4.83454143	–2.46685815
*N*	0.00021582	–0.00522535	0.05166750	–0.27058072	0.80759364	–1.35121423	1.76762350
Δln *A*	–0.00162360	0.03007617	–0.23026602	0.94553128	2.28459957	3.59240897	–2.05152007
*Q*^*A*^	0	0	–0.00142679	0.01530547	–0.03477028	1.10201529	–0.08060157

**Figure 13:**
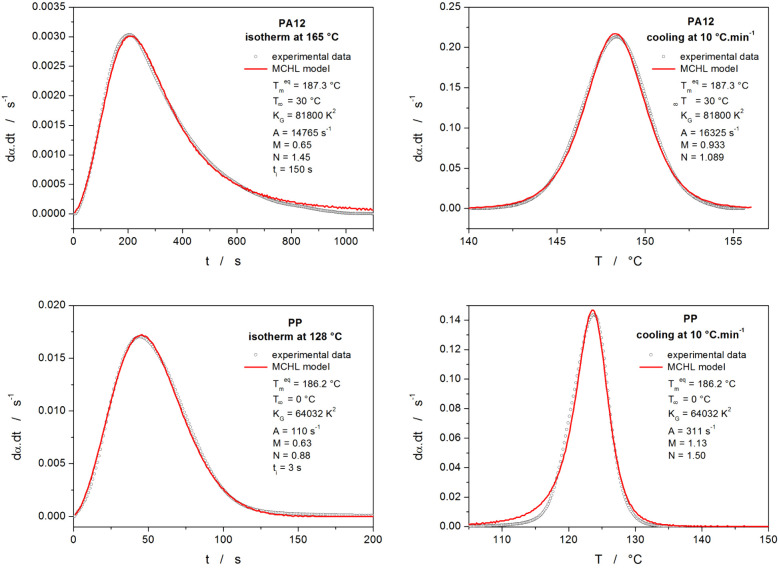
Examples of the isothermal and nonisothermal crystallization data obtained for polyamide 12 (PA12) and isotactic polypropylene (PP) from Figure 1 fit by the MCHL model (combination of eqs 2 and 9). The parameters of the best fits are displayed in the respective legends in each graph.

The second way of utilizing the accuracy of the MCHL model description of the polymer crystallization is via kinetic predictions. Historically, kinetic predictions have been the goal of kinetic analysis. This is even more true nowadays, when both the accuracy of the experimental instruments and the mathematical apparatus have progressed so that complete isolation of the true kinetic (crystallization) signal is possible (see, e.g., refs^55−57^ for the recent advancements in this field). By achieving an accurate (even though not necessarily physically meaningful) description of such data, extrapolation of the observed trends in the crystallization kinetics is then possible for the experimental conditions, where the measurements would be extremely difficult or impossible (e.g., due to the detection limits, baseline drift stability, minimum thermal gradients in the system, experimental time limitations, etc.). For successful and precise kinetic predictions, the accuracy of the primary data description is crucial.^58−60^ The flexibility of the present MCHL model represents an ultimate solution in this regard. This is demonstrated in Figure 13, where the experimental data from Figure 1 fit the MCHL model. Considering the worldwide recognized universality of eq 9 (with implemented Arrhenian rate constant kinetics) in the field of ceramics and glass-ceramics,^61−63^ its utilization for the description of the polymer crystallization paired with the Hoffman–Lauritzen description of the microscopic nucleation–growth rates can be deemed to cover practically all physico-chemically fundamental single-process crystallization mechanisms. Therefore, any incompatible experimental data should be described in terms of the complex-process kinetics,^58,59,64^ where the deconvolution of the crystallization signal is necessary for both mathematical and physicochemical description of the crystallization process.

## 3 Conclusions

Theoretical simulations were used to demonstrate that the Avrami-Hoffman–Lauritzen (AHL) concept is characterized by a strict asymmetry of the derivative (dα·d*t*^–1^) kinetic peaks (identical to the standard calorimetric crystallization signals of polymers). Whereas this asymmetry can change (even switch to the opposite values) for the nonisothermal crystallization peaks obtained at high heating rates, when the crystallization process shifts into the temperature region beyond the onset inflection point of the rate constant (*K*) temperature dependence, such behavior is still rather rare and limited to the experimental conditions that are uncommon. Moreover, under isothermal conditions, the Avrami theory does not allow for any change in the crystallization peak asymmetry. However, these findings are inconsistent with numerous experimental observations of the polymer crystallization process, for which, to date, no adequate mathematical modeling was properly developed, analyzed, and tested.

To overcome this limitation for advanced kinetics calculations, two autocatalytic models based on the broadly accepted Hoffman–Lauritzen theory combined with the *N*^th^-order kinetics were introduced and extensively analyzed in the present paper. Whereas the ZCHL model is more favorable concerning the physicochemical interpretation of its kinetic parameters, it fails to accurately reproduce the baseline AHL kinetics—and thus cannot be considered a more flexible and suitable alternative to the Avrami approach. On the other hand, the MCHL model not only precisely reproduces the AHL behavior but also covers a full range of the asymmetries experimentally observed for the polymer crystallization (tested for both nonisothermal and isothermal crystallization data). The kinetic peaks produced/following the MCHL model also comply with the *t*_0.5_-based evaluation of the Hoffman–Lauritzen nucleation constant (*K*_g_), further extending the practical utilizability of the MCHL model. Since both kinetic exponents (*M* and *N*) of the MCHL model influence the shape and asymmetry of the crystallization peaks uniquely and independently, their physicochemical interpretation is unambiguous. Furthermore, the direct correlation between the AHL and MCHL kinetic exponents’ values allows for advanced interpretations of the potential deviations of the crystallization behavior from the standard Avrami kinetics. Overall, the demonstrated flexibility and physicochemical interpretability of the introduced MCHL crystallization model leads to a conclusion that this model surpasses, and could fully replace, the present golden standard of the Avrami-Hoffman–Lauritzen approach.

## Data Availability

Data sets generated during the study are available at https://doi.org/10.6084/m9.figshare.28726550.v1.

## Supporting Information Available

ZCHL kinetic peaks simulated for the HL parameters of set A fromTable 1; fits of the different AHL kinetic peaks by the ZCHL model; fits of the different AHL kinetic peaks by the MCHL model; unzoomed Figure 7C; and differential and integral forms of the AHL model (PDF)

## References

[ref1] Zhang M. C.; Guo B. H.; Xu J. A review on polymer crystallization theories. Crystals 2017, 7, 4 10.3390/cryst7010004.

[ref2] Keller A.; Goldbeck-Wood G.; Hikosaka M. Polymer crystallization: survey and new trends with wider implications for phase transformations. Faraday Discuss 1993, 95, 109–128. 10.1039/fd9939500109.

[ref3] Sangroniz L.; Cavallo D.; Müller A. J. Self-nucleation effects on polymer crystallization. Macromolecules 2020, 53 (12), 4581–4604. 10.1021/acs.macromol.0c00223.

[ref4] Raimo M. Impact of thermal properties on crystalline structure, polymorphism and morphology of polymer matrices in composites. Materials 2021, 14, 2136 10.3390/ma14092136. 33922297 PMC8122767

[ref5] Gao P.; Masato D. The Effects of Nucleating Agents and Processing on the Crystallization and Mechanical Properties of Polylactic Acid: A Review. Micromachines 2024, 15, 776 10.3390/mi15060776. 38930746 PMC11206032

[ref6] Wunderlich B.; Davidson T. Extended-chain crystals. I. General crystallization conditions and review of pressure crystallization of polyethylene. J. Polym. Sci. A-2: Polym. Phys. 1969, 7, 2043–2050. 10.1002/pol.1969.160071206.

[ref7] Johnson W. A. Reaction kinetics in process of nucleation and growth. Trans. Am. Inst. Min. Metall. Eng. 1939, 135, 416–458.

[ref8] Avrami M. Kinetics of phase change. I General theory. J. Chem. Phys. 1939, 7, 1103–1112. 10.1063/1.1750380.

[ref9] Avrami M. Kinetics of phase change. II transformation-time relations for random distribution of nuclei. J. Chem. Phys. 1940, 8, 212–224. 10.1063/1.1750631.

[ref10] Avrami M. Granulation, phase change, and microstructure kinetics of phase change. III. J. Chem. Phys. 1941, 9, 177–184. 10.1063/1.1750872.

[ref11] Kolmogorov A. N. On the statistical theory of the crystallization of metals. Bull. Acad. Sci. URSS (Cl. Sci. Math. Nat.) 1937, 1, 335–359.

[ref12] Hoffman J. D.; Davis G. T.; Lauritzen J. I. Jr. The rate of crystallization of linear polymers with chain folding Treatise on Solid State Chemistry, (Crystalline and Noncrystalline Solids) Hannay N. B.Ed.Springer Boston, MA 1976 3 497–614 10.1007/978-1-4684-2664-9_7

[ref13] Huang T.; Zhou C. Surface Diffusion-Controlled Jonhson–Mehl–Avrami–Kolmogorov Model for Hydrogenation of Mg-based Alloys. J. Phys. Chem. C 2023, 127, 13900–13910. 10.1021/acs.jpcc.3c02650.

[ref14] Pang Y.; Sun D.; Gu Q.; Chou K. C.; Wang X.; Li Q. Comprehensive determination of kinetic parameters in solid-state phase transitions: an extended Jonhson–Mehl–Avrami–Kolomogorov model with analytical solutions. Cryst. Growth Des. 2016, 16, 2404–2415. 10.1021/acs.cgd.6b00187.

[ref15] Frank F. C.; Tosi M. On the theory of polymer crystallization. Proc. R. Soc. London, Ser. A 1961, 263, 323–339. 10.1098/rspa.1961.0163.

[ref16] Lauritzen J. I. Jr.; Passaglia E. Kinetics of crystallization in multicomponent systems. II. Chain-folded polymer crystals. J. Res. Natl. Bur. Stand. Sec. A 1967, 71A (4), 261–275. 10.6028/jres.071A.033. PMC6658453 31824052

[ref17] Hoffman J. D.; Miller R. L. Surface nucleation theory for chain-folded systems with lattice strain: curved edges. Macromolecules 1989, 22, 3038–3054. 10.1021/ma00197a027.

[ref18] Point J. J.; Villers D. Nucleation-controlled growth and normal growth: a unified view. J. Cryst. Growth 1991, 114, 228–238. 10.1016/0022-0248(91)90696-3.

[ref19] Hoffman J. D.; Miller R. L. Response to criticism of nucleation theory as applied to crystallization of lamellar polymers. Macromolecules 1989, 22, 3502–3505. 10.1021/ma00198a055.

[ref20] Hoffman J. D. Transition from extended-chain to once-folded behaviour in pure n-paraffins crystallized from the melt. Polymer 1991, 32, 2828–2841. 10.1016/0032-3861(91)90116-Z.

[ref21] Hoffman J. D. The relationship of C∞ to the lateral surface free energy σ: estimation of C∞ for the melt from rate of crystallization data. Polymer 1992, 33, 2643–2644. 10.1016/0032-3861(92)91149-V.

[ref22] Sadler D. M.; Gilmer G. H. A model for chain folding in polymer crystals: rough growth faces are consistent with the observed growth rates. Polymer 1984, 25, 1446–1452. 10.1016/0032-3861(84)90108-3.

[ref23] Sadler D. M. Roughness of growth faces of polymer crystals: Evidence from morphology and implications for growth mechanisms and types of folding. Polymer 1983, 24, 1401–1409. 10.1016/0032-3861(83)90220-3.

[ref24] Sadler D. M.; Gilmer G. H. Rate-theory model of polymer crystallization. Phys. Rev. Lett. 1986, 56, 2708 10.1103/PhysRevLett.56.2708. 10033070

[ref25] Hikosaka M. Unified theory of nucleation of folded-chain crystals (FCCs) and extended-chain crystals (ECCs) of linear-chain polymers: 2. Origin of FCC and ECC. Polymer 1990, 31, 458–468. 10.1016/0032-3861(90)90385-C.

[ref26] Hikosaka M. Unified theory of nucleation of folded-chain crystals and extended-chain crystals of linear-chain polymers. Polymer 1987, 28, 1257–1264. 10.1016/0032-3861(87)90434-4.

[ref27] Hikosaka M.; Amano K.; Rastogi S.; Keller A. Crystallization of Polymers; Kluwer Academic Publishers: Dordrecht, Netherlands, 1993; p 331.

[ref28] Hikosaka M.; Watanabe K.; Okada K.; Yamazaki S. Topological mechanism of polymer nucleation and growth–the role of chain sliding diffusion and entanglement. In Interphases and mesophases in polymer crystallization III, Allegra G., Ed.; Springer, 2005.

[ref29] Wunderlich B.; Mehta A. Macromolecular nucleation. J. Polym. Sci. Polym. Phys. Ed. 1974, 12, 255–263. 10.1002/pol.1974.180120203.

[ref30] Mehta A.; Wunderlich B. A study of molecular fractionation during the crystallization of polymers. Colloid Polym. Sci. 1975, 253, 193–205. 10.1007/BF01470229.

[ref31] Cheng S. Z.; Noid D. W.; Wunderlich B. Molecular segregation and nucleation of poly (ethylene oxide) crystallized from the melt. IV. Computer modeling. J. Polym. Sci., Part B: Polym. Phys. 1989, 27, 1149–1160. 10.1002/polb.1989.090270514.

[ref32] Hu W.; Frenkel D.; Mathot V. B. Intramolecular nucleation model for polymer crystallization. Macromolecules 2003, 36, 8178–8183. 10.1021/ma0344285.

[ref33] Hu W.; Cai T. Regime transitions of polymer crystal growth rates: molecular simulations and interpretation beyond Lauritzen-Hoffman model. Macromolecules 2008, 41, 2049–2061. 10.1021/ma702636g.

[ref34] Kundagrami A.; Muthukumar M. Continuum theory of polymer crystallization. J. Chem. Phys. 2007, 126 (14), 144901 10.1063/1.2713380. 17444737

[ref35] Leung W. M.; John Manley R. S.; Panaras A. R. Isothermal growth of low molecular weight polyethylene single crystals from solution. 3. Macromolecules 1985, 18, 760–771. 10.1021/ma00146a031.

[ref36] Cheng S. Z.; Chen J.; Heberer D. P. Extended chain crystal growth of low molecular mass poly (ethylene oxide) and α, ω-methoxy poly (ethylene oxide) fractions near their melting temperatures. Polymer 1992, 33, 1429–1436. 10.1016/0032-3861(92)90118-G.

[ref37] Shirzad K.; Viney C. A critical review on applications of the Avrami equation beyond materials science. J. R. Soc., Interface 2023, 20 (203), 20230242 10.1098/rsif.2023.0242. 37340781 PMC10282574

[ref38] Blázquez J. S.; Romero F. J.; Conde C. F.; Conde A. A review of different models derived from classical Kolmogorov, Johnson and Mehl, and Avrami (KJMA) theory to recover physical meaning in solid-state transformations. Phys. Status Solidi B 2022, 259, 2100524 10.1002/pssb.202100524.

[ref39] Ozawa T. Kinetics of non-isothermal crystallization. Polymer 1971, 12, 150–158. 10.1016/0032-3861(71)90041-3.

[ref40] Jeziorny A. Parameters characterizing the kinetics of the non-isothermal crystallization of poly (ethylene terephthalate) determined by DSC. Polymer 1978, 19, 1142–1144. 10.1016/0032-3861(78)90060-5.

[ref41] Liu T.; Mo Z.; Wang S.; Zhang H. Nonisothermal melt and cold crystallization kinetics of poly (aryl ether ether ketone ketone. Polym. Eng. Sci. 1997, 37, 568–575. 10.1002/pen.11700.

[ref42] Nakamura K.; Watanabe T.; Katayama K.; Amano T. Some aspects of nonisothermal crystallization of polymers. I. Relationship between crystallization temperature, crystallinity, and cooling conditions. J. Appl. Polym. Sci. 1972, 16, 1077–1091. 10.1002/app.1972.070160503.

[ref43] Delogu F.; Cocco G. Kinetics of amorphization processes by mechanical alloying: a modeling approach. J. Alloys Compd. 2007, 436, 233–240. 10.1016/j.jallcom.2006.07.029.

[ref44] Delogu F.; Deidda C.; Mulas G.; Schiffini L.; Cocco G. A quantitative approach to mechanochemical processes. J. Mater. Sci. 2004, 39, 5121–5124. 10.1023/B:JMSC.0000039194.07422.be.

[ref45] Delogu F.; Takacs L. Mechanochemistry of Ti–C powder mixtures. Acta Mater. 2014, 80, 435–444. 10.1016/j.actamat.2014.08.036.

[ref46] Pérez-Ćardenas F. C.; Castillo L. F. D.; Vera-Graziano R. Modified Avrami expression for polymer crystallization kinetics. J. Appl. Polym. Sci. 1991, 43, 779–782. 10.1002/app.1991.070430416.

[ref47] Cahn J. W. The kinetics of grain boundary nucleated reactions. Acta Metall. 1956, 4, 449–459. 10.1016/0001-6160(56)90041-4.

[ref48] Zhang Z.; Shao Y.; Huang L.; Liu X.; Han F.; Yan P. A new hydration kinetic model based on boundary nucleation and growth mechanism with time-dependent growth rate: Application to quantitively characterize the influence of alkali on the early hydration of cement, Constr. Build. Mater. 2024, 411, 134616 10.1016/j.conbuildmat.2023.134616.

[ref49] Svoboda R. Johnson-Mehl-Avrami kinetics as a universal description of crystallization in glasses?. J. Eur. Ceram. Soc. 2024, 44, 4064–4082. 10.1016/j.jeurceramsoc.2023.12.096.

[ref50] Luciano G.; Soetaert K.; Svoboda R. Simulation and non-linear optimization of Šesták-Berggren kinetics. J. Non-Cryst. Solids 2020, 550, 120391 10.1016/j.jnoncrysol.2020.120391.

[ref51] Luciano G.; Svoboda R. Simulation and non-linear optimization of kinetic models for solid-state processes, Model. Simul. Mater. Sci. Eng. 2024, 32, 035014 10.1088/1361-651X/ad2788.

[ref52] Svoboda R.; Zmrhalová Z. O.; Galusek D.; Brandová D.; Chovanec J. Thermal decomposition of mixed calcium oxalate hydrates – kinetic deconvolution of complex heterogeneous processes. Phys. Chem. Chem. Phys. 2020, 22 (16), 8889–8901. 10.1039/C9CP06867H. 32289819

[ref53] Svoboda R. Fraser-Suzuki function as an essential tool for mathematical modeling of crystallization in glasses. J. Eur. Ceram. Soc. 2024, 44, 401–407. 10.1016/j.jeurceramsoc.2023.08.050.

[ref54] Šesták J. Thermophysical properties of solids: their measurements and theoretical thermal analysis; Elsevier: Amsterdam, 1984.

[ref55] Svoboda R. Importance of proper baseline identification for the subsequent kinetic analysis of derivative kinetic data: Part 3. J. Therm. Anal. Calorim. 2019, 136, 1307–1314. 10.1007/s10973-018-7738-1.

[ref56] Svoboda R.; Maqueda L. P.; Podzemná V.; Perejon A.; Svoboda O. Influence of DSC thermal lag on evaluation of crystallization kinetics. J. Non-Cryst. Solids 2020, 528, 119738 10.1016/j.jnoncrysol.2019.119738.

[ref57] Svoboda R. Extended theoretical analysis of crystallisation kinetics being studied by in situ XRD. Philos. Mag. 2020, 100, 713–727. 10.1080/14786435.2019.1704901.

[ref58] Brandová D.; Svoboda R.; Zmrhalová Z. O.; Chovanec J.; Bulánek R.; Romanová J. Crystallization kinetics of glassy materials: the ultimate kinetic complexity?. J. Therm. Anal. Calorim. 2018, 134, 825–834. 10.1007/s10973-018-7078-1.

[ref59] Svoboda R.; Chovanec J.; Šlang S.; Beneš L.; Konrad P. Single-curve multivariate kinetic analysis: Application to the crystallization of commercial Fe-Si-Cr-B amorphous alloys. J. Alloys Compd. 2021, 889, 161672 10.1016/j.jallcom.2021.161672.

[ref60] Svoboda R. Thermal decomposition of active pharmaceutical substances and accuracy of the related kinetic predictions: The case of nifedipine. Thermochim. Acta 2024, 738, 179790 10.1016/j.tca.2024.179790.

[ref61] Šesták J.; Berggren G. Study of the kinetics of the mechanism of solid-state reactions at increasing temperatures. Thermochim. Acta 1971, 3, 1–12. 10.1016/0040-6031(71)85051-7.

[ref62] Šesták J. Šesták–Berggren equation: now questioned but formerly celebrated-what is right. J. Therm. Anal. Calorim. 2017, 127, 1117–1123. 10.1007/s10973-015-4998-x.

[ref63] Šimon P. Fourty years of the Šesták–Berggren equation. Thermochim. Acta 2011, 520, 156–157. 10.1016/j.tca.2011.03.030.

[ref64] Svoboda R.; Málek J. Applicability of Fraser–Suzuki function in kinetic analysis of complex crystallization processes. J. Therm. Anal. Calorim. 2013, 111, 1045–1056. 10.1007/s10973-012-2445-9.

