# Correction to “Polymer Crystallization: Universal Macroscopic Description via the Autocatalytic Hoffman–Lauritzen Approach”

**DOI:** 10.1021/acsomega.5c10235

**Published:** 2025-11-11

**Authors:** Roman Svoboda, Jana Machotová

We regret to say that during our literature search on the subject, we overlooked a prior contribution by Sbirrazzuoli [^1^], who had already utilized the combination of eqs 2 + 9 in description of crystallization of poly­(ethylene 2,5-furandicarboxylate). His work provides a clear precedent and should be acknowledged for its relevance to our findings.
