# Supplementary material for: Polymer Crystallization: Universal Macroscopic Description via the Autocatalytic Hoffman–Lauritzen Approach
Source: ACS Omega. 2025 Apr 20;10(16):16602–19. doi: 10.1021/acsomega.5c00067 (PMC12044480; doi:10.1021/acsomega.5c00067)
Supplement: Supplementary file 1 — ao5c00067_si_001.pdf [file ao5c00067_si_001.pdf]

Supporting information

Polymer crystallization: Universal macroscopic description via autocatalytic Hoffman-Lauritzen approach

Roman Svoboda [a], Jana Machotová [b,*]

[a] *Department of Physical Chemistry, Faculty of Chemical Technology, University of Pardubice, Studentská 573, 532 10 Pardubice, Czech Republic.*

[b] *Institute of Chemistry and Technology of Macromolecular Materials, Faculty of Chemical Technology, University of Pardubice, Studentská 573, 532 10 Pardubice, Czech Republic.*

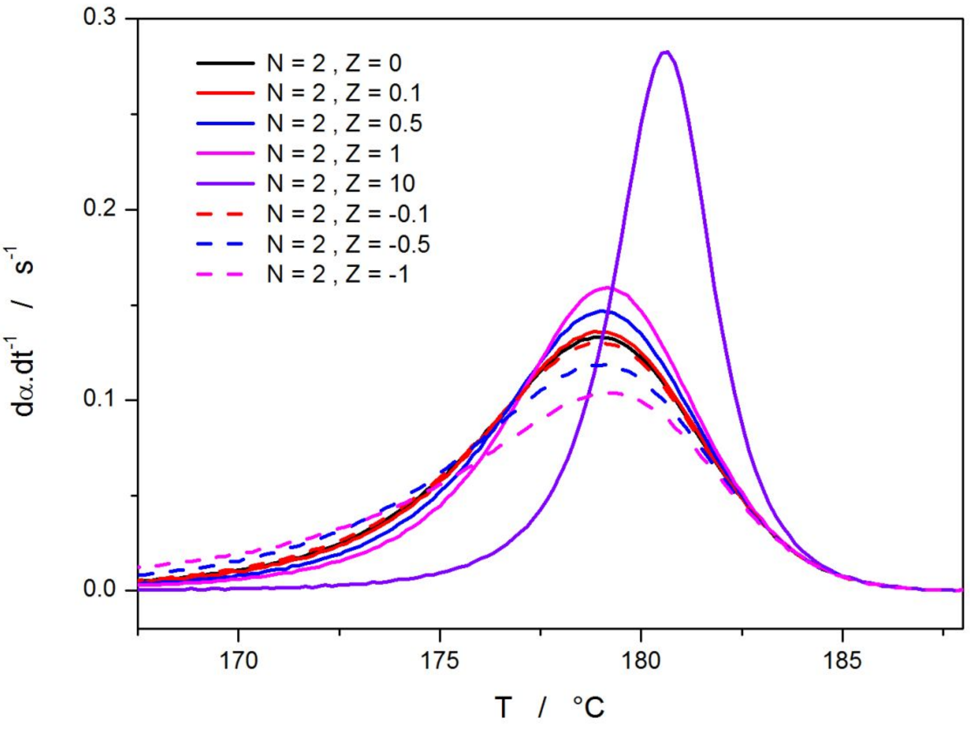

Fig. S1. Set of ZCHL kinetic peaks simulated for the HL parameters of set A from Table 1 and q^{-} = 1 °C·min^{-1} and N = 2.

* Corresponding author: jana.machotova@upce.cz

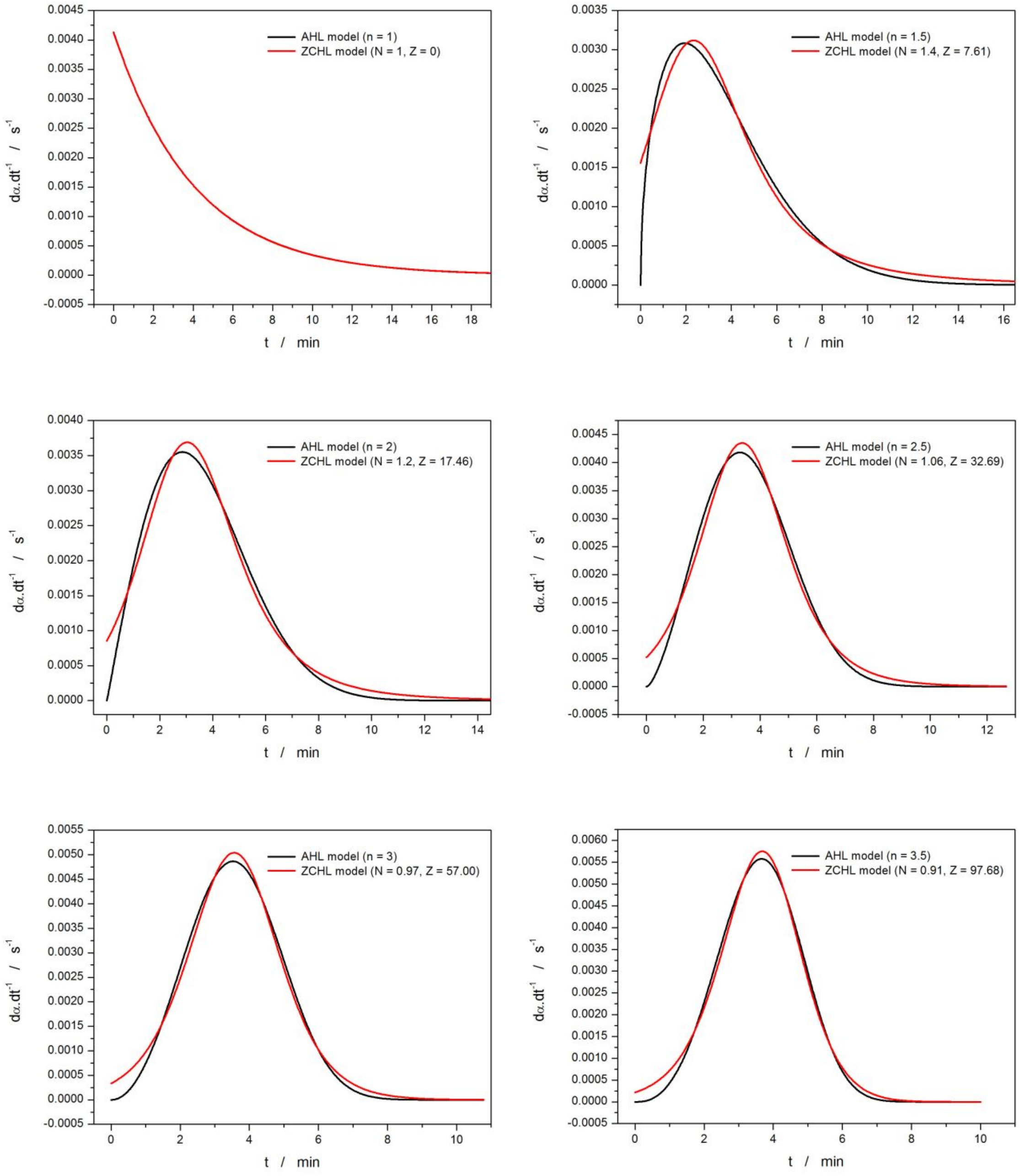

Fig. S2. Best fits of the different AHL kinetic peaks (with varying *n*) by the ZCHL model – the optimized ZCHL kinetic exponent are listed in the graphs‘ legends.

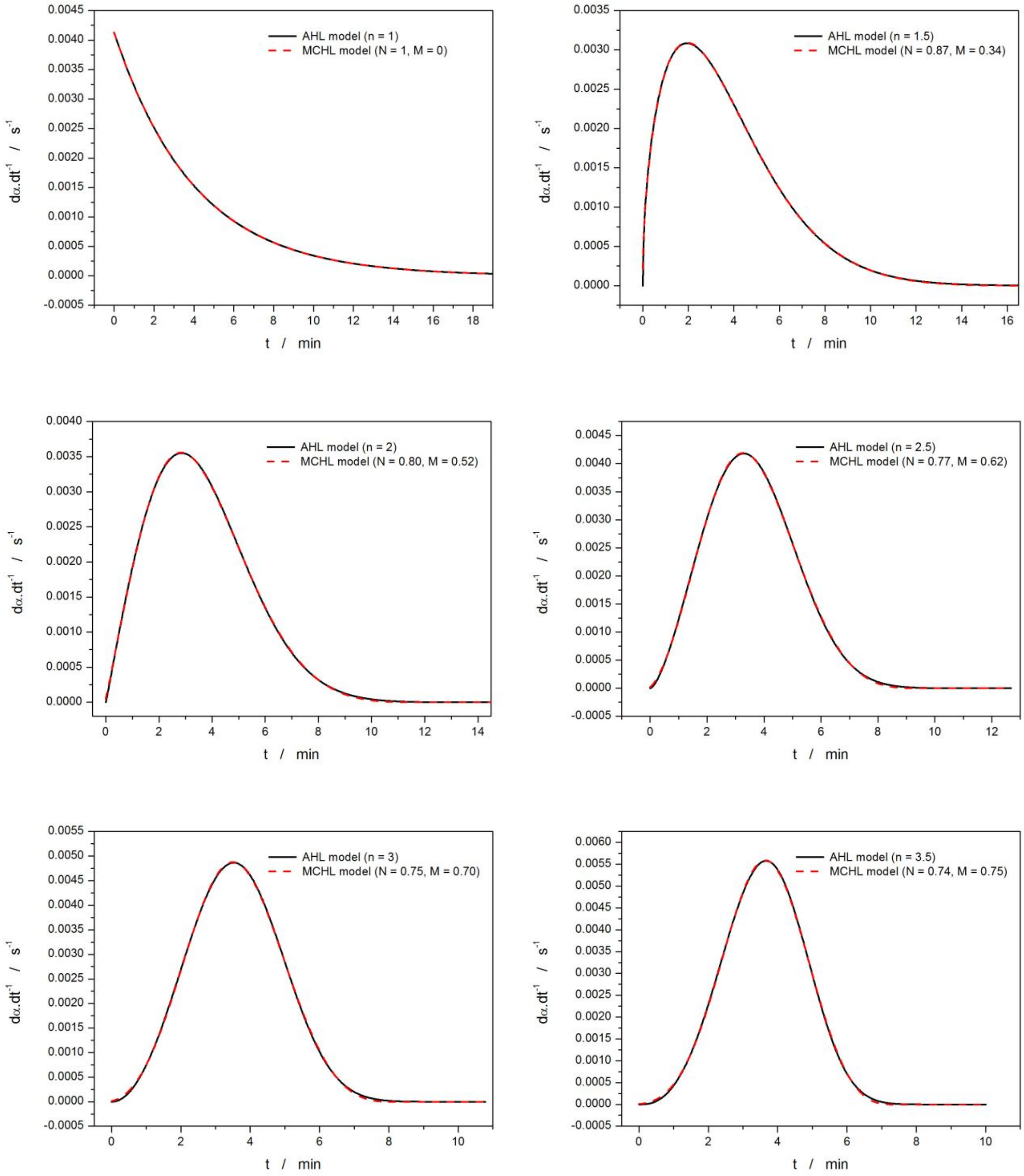

Fig. S3. Best fits of the different AHL kinetic peaks (with varying *n*) by the MCHL model – the optimized MCHL kinetic exponent are listed in the graphs‘ legends.

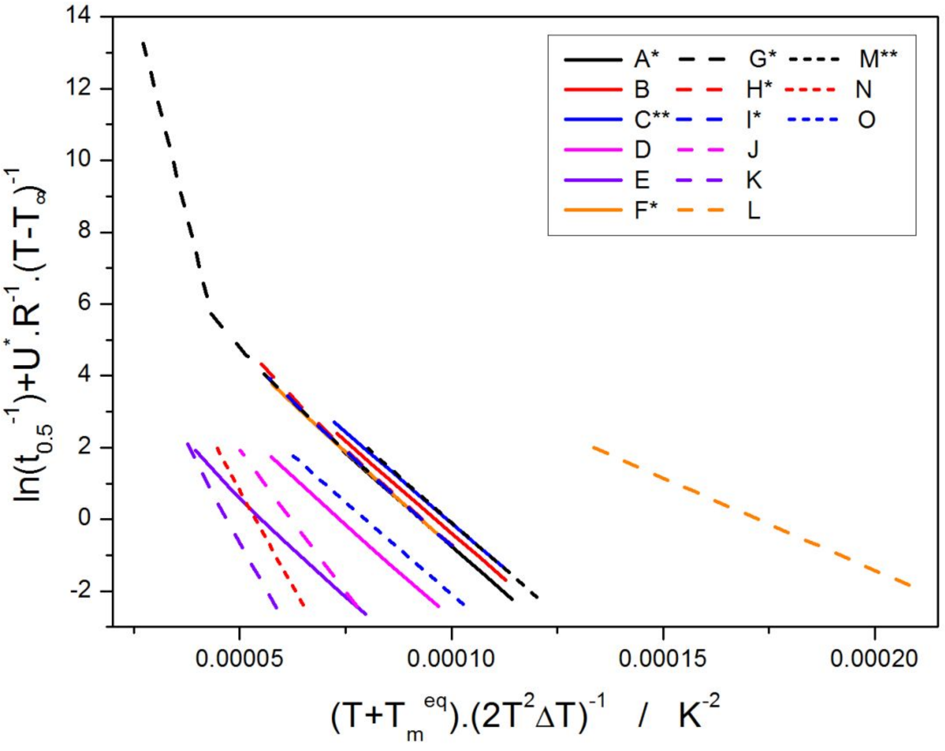

Fig. S4. Un-zoomed **Fig. 7**c showing the curvature of the dependence corresponding to the G dataset.

Equation S1 and **S2** represent the combination of **Equation 1** and **2** used for the simulation of the AHL model.

The differential form:

$$\frac{d\alpha}{dt} = \frac{d\left(1-exp\left(-\left\{\int_0^t \left(A \cdot exp\left(-\frac{U}{R(T-T\infty)}\right)exp\left(-\frac{K_G}{T\Delta Tf}\right)\right)dt\right\}^n\right)\right)}{dt} \qquad \text{(S1)}$$

The integral form:

$$\alpha(t) = 1 - exp\left(-\left\{\int_0^t \left(A \cdot exp\left(-\frac{U}{R(T-T_\infty)}\right)exp\left(-\frac{K_G}{T\Delta Tf}\right)\right)dt\right\}^n\right) \qquad \text{(S2)}$$

For non-isothermal conditions, T and ΔT are functions of t (linear cooling from T_m to T_∞ for the present paper).
